# Infection vs. Reinfection: The Immunomodulation of Erythropoiesis

**DOI:** 10.3390/ijms25116153

**Published:** 2024-06-03

**Authors:** Ana Catarina Pêgo, Illyane Sofia Lima, Ana Catarina Martins, Inês Sá-Pereira, Gracelino Martins, Raffaella Gozzelino

**Affiliations:** NOVA Medical School Research, NOVA University of Lisbon, 1150-082 Lisbon, Portugal

**Keywords:** erythropoiesis, immunity, infection, severe malarial anemia, iron metabolism

## Abstract

Severe malarial anemia (SMA) increases the morbidity and mortality of *Plasmodium*, the causative agent of malaria. SMA is mainly developed by children and pregnant women in response to the infection. It is characterized by ineffective erythropoiesis caused by impaired erythropoietin (EPO) signaling. To gain new insights into the pathogenesis of SMA, we investigated the relationship between the immune system and erythropoiesis, conducting comparative analyses in a mouse model of malaria. Red blood cell (RBC) production was evaluated in infected and reinfected animals to mimic endemic occurrences. Higher levels of circulating EPO were observed in response to (re)infection. Despite no major differences in bone marrow erythropoiesis, compensatory mechanisms of splenic RBC production were significantly reduced in reinfected mice. Concomitantly, a pronounced immune response activation was observed in erythropoietic organs of reinfected animals in relation to single-infected mice. Aged mice were also used to mimic the occurrence of malaria in the elderly. The increase in symptom severity was correlated with the enhanced activation of the immune system, which significantly impaired erythropoiesis. Immunocompromised mice further support the existence of an immune-shaping regulation of RBC production. Overall, our data reveal the strict correlation between erythropoiesis and immune cells, which ultimately dictates the severity of SMA.

## 1. Introduction

Malaria is an ancient disease that has been threatening humans for thousands of years. A protozoan parasite, from the *Plasmodium* (*P*.) genus family, is responsible for the infection. More than 120 species of *Plasmodium* have been identified, but only five infect humans [1]. Among those, the prevalence of *P. falciparum* is associated with the higher morbidity and mortality rates of malaria in tropical regions, where it geographically spreads [2,3].

Over 247 million cases have been reported worldwide [4]. Despite advances in disease surveillance, treatment, and patient monitoring programs, that number continues to increase. Malaria is responsible for the death of millions of people, causing about 620,000 fatalities per year [5], one-third of which occur in Sub-Saharan Africa [2,6]. Children under 5 years of age are mostly affected and account for 80% of all *Plasmodium*-induced deaths [7]. In endemic areas, this threat is aggravated by multiple malaria exposures often faced by the local population. This leads to recurrent malaria, classified in three phases according to episodes of recrudescence, relapse, or reinfection. Recrudescence is associated with an incomplete parasite clearance that causes the reappearance of malaria symptoms. It is often provoked by treatment failure, presumably induced by parasite resistance. Relapse is triggered by dormant parasites, reactivated upon an initial infection clearance. Lastly, reinfection occurs when new mosquito bites affect individuals that have been previously exposed to malaria [8]. In endemic regions, those patients elicit an exacerbated immune response regardless of their age. Evidence at this regard have been collected also when evaluating infected children [9]. In mice, reinfection models allow us to better understand and investigate the development of severe malaria anemia (SMA) [10,11].

SMA is one of the leading causes of death by *Plasmodium* infection [2,12]. In children, SMA induces 5.6% to 16% mortality. The World Health Organization defines SMA as the condition in which the hemoglobin level decreases below 5 g/dl or the hematocrit below 15%. While those values apply to young individuals, aged 12 years or less, SMA also causes 6% mortality in pregnant women. It affects primigravidae, especially [13]. In adults, SMA occurs when the hemoglobin level is lower than 7 g/dL and hematocrits are less than 20% [14]. SMA is difficult to diagnose and mainly develops when parasitemia is higher than 10,000/µL. In some cases, it manifests upon *Plasmodium* clearance [15] due to the enhanced phagocytic activity of the reticuloendothelial system. The red blood cell (RBC) count decreases [16], and the high degree of hemolysis, caused by the proliferation of *Plasmodium* inside RBCs, impairs erythropoiesis [12,17,18].

One of the main features of SMA is the inability to activate a proper erythrocytic response [19] regardless of erythropoietin (EPO) levels. EPO is a hormone, synthesized by the kidney, responsible for the proliferation and differentiation of erythroid cells. It is induced by hypoxia [20,21], which, in turn, is caused by hemolysis. The role of EPO in malaria infection is still controversial, considering that non-infected and infected individuals present similar circulating levels of this hormone [22]. This suggests that a decreased concentration of EPO is unlikely to play a major role in SMA, which is also supported by the notion that, in malaria, circulating EPO is higher when compared to other pathologies causing the same degree of anemia [23]. Nevertheless, *Plasmodium*-infected patients continue to suffer from low reticulocytosis and reduced RBC counts, suggesting the interference of other mechanisms regulating erythroid cell proliferation. It has been well established that hypoxia underlies the development of SMA and that it is influenced by the activation of the immune system. The inflammatory mediators released by the host to cope with the infection negatively affect erythropoiesis [24,25]. However, in case of SMA, the reported clinical symptoms seem to occur regardless of the levels of pro-inflammatory cytokines released into circulation [26,27]. This leads us to hypothesize that toxic metabolites produced by malaria parasites or released into the bloodstream upon infection, like hemozoin [28], might play a role in this controversy. This byproduct, generated via hemoglobin digestion, is likely associated with an increase in EPO production and effectivity during malaria [26,27]. In mice, EPO stimulation has been shown to improve animal survival in response to malaria, enhancing RBC production and reducing hypoxia, iron (Fe) cytotoxicity, and tissue damage. In agreement, the pharmacological inhibition of EPO has been found to increase their mortality, indicating that reestablishing erythropoiesis is a crucial step to recover from SMA [22].

The aim of this study was to evaluate the cross-talk between immunity and erythropoiesis, in response to malaria, to shed light regarding the pathogenesis of an infection that, after millennia, remains still poorly understood. Hence, comparative analyses were conducted in infected and reinfected mice, also assessing the influence of aging. The impact of the immune system on erythropoiesis was further confirmed by using immune-deficient animals, deprived of the adaptive immune system [29], which allowed us to reveal potential targets to be deeply investigated in the future.

## 2. Results

### 2.1. Changes in EPO Signaling in Infected and Reinfected Mice

Erythropoiesis is a tightly regulated process. It is induced by EPO [30]. Produced by the kidney and released into circulation, EPO migrates to the bone marrow, where it binds to the EPO receptor (EPO-R) on precursor cells [31]. By promoting the expansion of erythroid lineages, EPO stimulates the proliferation and differentiation of erythroid progenitors into mature erythrocytes [30]. Iron availability is, therefore, the limiting factor in this process [32].

**Figure 1:**
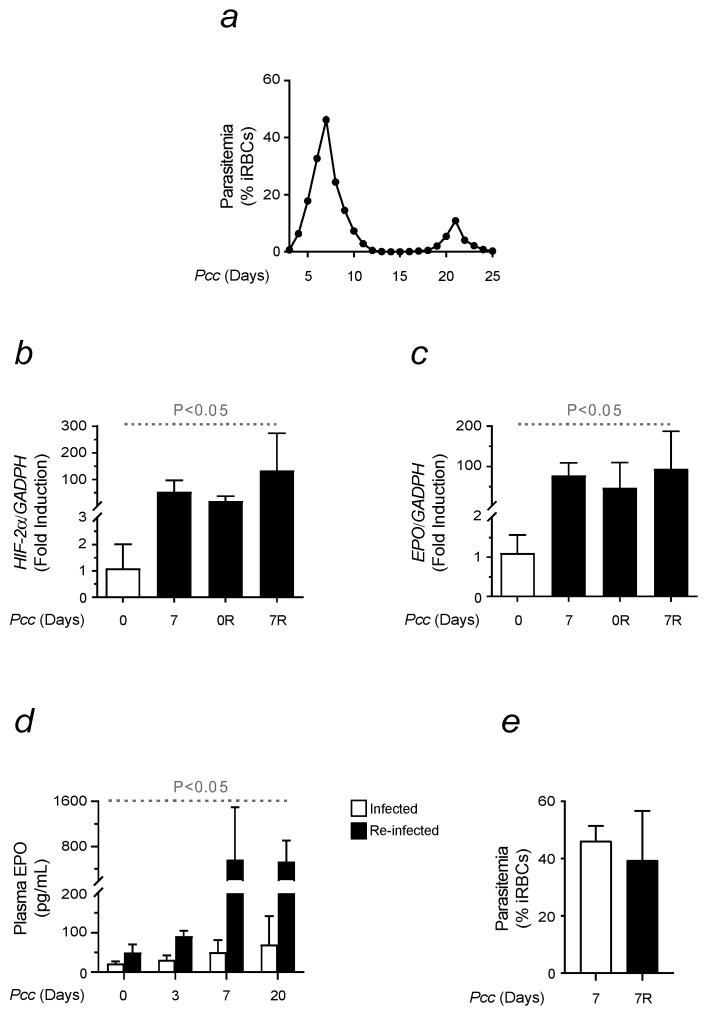
Changes in EPO signaling in *Pcc*-infected and reinfected mice. C57BL/6 mice, aged between 8 and 12 weeks, were injected i.p. with 10^6^ *Pcc*-infected RBCs. Reinfected animals were left to recover for a period of 60 days before being newly infected with the same strain of parasite. (**a**) Mean parasitemia were measured from day 0 to day 25 post-infection, through the Giemsa staining of blood smears (n = 10). (**b**) mRNA expression of *HIF-2α*, quantified in the kidney in infected and reinfected mice on day 0 and day 7 (n = 6–8). (**c**) mRNA expression of *EPO*, quantified as in (**b**) (n = 6–8). (**d**) Plasma EPO in infected and reinfected mice, expressed in pg/mL for indicated time points (n = 6–8). (**e**) Parasitemia counted in blood smears of infected and reinfected (R) mice, as assessed in (**a**) on day 7 (n = 4–10). Data are presented as mean values ± standard deviations. Statistically significant differences were calculated via one-way ANOVA. When indicated, significancy refers to the differences that were found when comparing the results of the time course of malaria between infected and reinfected mice, as well as the time point referred to for each condition.

Ineffective erythropoiesis is a common complication of malaria and other hemolytic disorders. It often occurs regardless of elevated EPO levels in circulation [22]. A certain degree of EPO unresponsiveness has also been found in malaria-infected mice. Aggravated by the replication of *Plasmodium* within the RBC compartment, this is known to compromise the capacity of BM to enhance erythropoiesis and rapidly recover from malaria [13,33,34]. The phenomenon was investigated in mice that were exposed to infection and reinfection conditions to mimic endemic occurrences. For this, C57BL/6 mice, aged between 8 and 12 weeks, were inoculated with 10^6^ *Plasmodium chabaudi chabaudi* (*Pcc* - *AS* strain) infected RBCs via intraperitoneal injection. All animals developed severe forms of malaria, characterized by exacerbated hemolysis, causing SMA. All *Pcc*-induced mice survived, clearing the infection within 4 weeks. Parasitemia was monitored daily through the Giemsa staining of blood smears. A peak of infection was consistently observed 7 days after *Pcc* inoculation, corresponding to the development of malaria symptoms. A lower peak was noticed 20 days later and preceded malaria resolution (Figure 1a). Mice were left to recover for a period of 2 months before being reinfected with the inoculating procedures previously described. Similarly, all animals survived the second exposure.

Comparative analyses were conducted to assess the erythropoietic response. The activation of EPO signaling was measured at the peak of parasitemia in infected and reinfected mice. The expression of the hypoxia-inducible factor (*HIF*)*-2α* gene was first quantified in the kidney using real-time PCR on day 7, i.e., when mice reached the maximum degree of hemolysis. Non-manipulated and recovered mice were used as controls. *HIF-2α* expression was normalized using *GADPH* as the housekeeping gene. Major differences were observed in both infected and reinfected animals, considering the significant increase in *HIF-2α* levels during malaria (Figure 1b). The expression of *EPO* followed the same trend, with reinfected animals showing an activated pathway already at the basal level (Figure 1c). When measuring the release of this hormone into circulation, a gradual increase in EPO levels was observed, as assessed via ELISA at indicated time points (Figure 1d). Higher concentrations of EPO were found in reinfected mice to be already at the basal level and increasing significantly during malaria (Figure 1d). No differences in parasitemia were found when infected and reinfected mice were compared on day 7 (Figure 1e).

### 2.2. Bone Marrow Erythropoietic Response in Infected and Reinfected Mice

**Figure 2:**
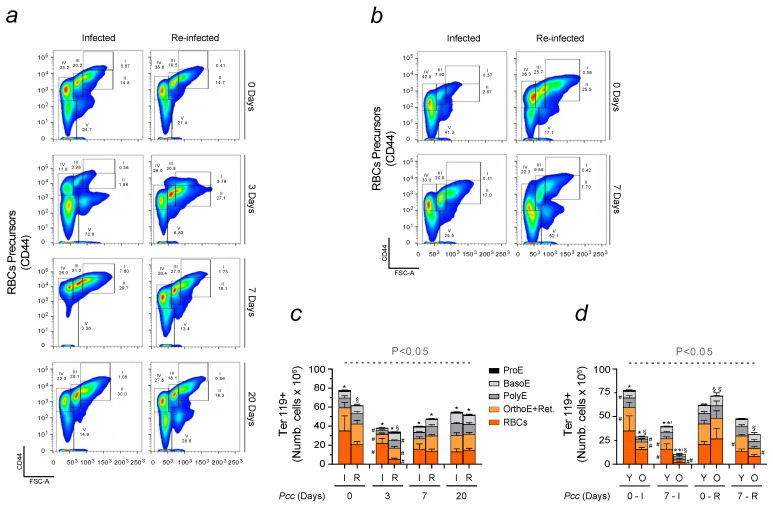
Erythroid lineage evaluation in infected and reinfected mice. (**a**) Gating strategy for erythroid cells in infected and reinfected mice on indicated days. Ter119+ cells were analyzed and, based on FSC-A and CD44, five subpopulations were identified. Proerythroblasts (I) showed higher expression of CD44 and FSC-A; basophilic erythroblasts (II), intermediate FSC-A and high CD44 expression; polychromatic erythroblasts (III), reduced FSC-A and high CD44 expression; orthochromatic erythroblasts and immature reticulocytes (IV), low FSC-A and high CD44 expression; and mature red blood cells (V), low FSC-A and CD44 expression. (**b**) Gating strategy to assess the different phases of erythropoiesis in infected and reinfected aged mice. (**c**) Flow cytometry quantification of graphs in (**a**), with histograms displaying the number of cells of each erythroid subpopulation: proerythroblast (ProE, black), basophilic (BasoE, light grey), polychromatic (PolyE, dark grey), orthochromatic and reticulocyte (OrthoE+Retic, yellow), and mature RBC (RBCs, orange). (**d**) Flow cytometry quantification of graphs in (**b**), comparing infected (I) and reinfected (R) young (Y) and old (O) mice. Data are presented as mean values ± standard deviations (n = 8–13). Statistically significant differences were calculated via one-way ANOVA. When indicated, significancy refers to the differences that were found when comparing the results of the time course of malaria between infected and reinfected mice. * refers to statistically significant data obtained when comparing non-manipulated mice to infected and reinfected animals at indicated time points. *’ refers to statistically significant data obtained when comparing infected and reinfected mice at specific time points. § refers to statistically significant data obtained when comparing recovered control mice to reinfected animals at indicated time points. §’ refers to statistically significant data obtained when comparing reinfected aged animals at specific time points. # refers to statistically significant data obtained when comparing indicated populations at indicated time points, between infected and reinfected conditions.

To better understand the dynamics of SMA, we evaluated erythropoiesis in infected and reinfected mice. The production of RBCs is induced in the bone marrow, upon the binding of EPO to its receptor EPOR on erythroid precursors [31]. The different stages of this process were assessed through flow cytometry. Ter119 was used as an erythroid marker of differentiation. Distinct populations were also identified using CD44, which decreased progressively during cell development, i.e., from proerythroblasts (ProEs) to reticulocytes (Retics). Representative graphs and their correspondent quantifications demonstrated that despite reduced RBC production in infected and reinfected mice at the basal level, major changes were observed 3 days after *Pcc* inoculation (Figure 2a). When assessing the different stages of erythropoiesis in aged mice, we observed that reinfected animals presented a diminished capacity to produce RBCs in relation to infected animals (Figure 2b). In response to malaria, ineffective erythropoiesis was found to be more exacerbated in aged mice than young mice, especially in the first phases of reinfection. Please note that all aged animals died before reaching day 20 post-reinfection. This finding was supported by the notion that age reduces resistance to malaria [35]. The number of Ter119+ cells was quantified and is shown here for all analyzed erythropoietic populations (Figure 2c,d). Inefficient erythropoiesis was more pronounced in reinfected mice, especially when assessing older animals, which presented lower counts already at the basal level. Contrarily to the single infection, both young and old animals were capable of enhancing erythropoiesis when reinfected. However, since this condition was associated with 100% lethality, the findings indicate that the severity of malaria in older mice was exacerbated.

To enable erythroid maturation, Fe is up-taken by precursor cells [36]. This is a limiting step for RBC production and was assessed first by quantifying the abundance of CD71, used as a marker for transferrin receptor 1 (TfR1). Significant differences in the number of CD71+ cells were observed among erythroid populations, with the ProE subset showing major differences at basal levels and at 3 days post *Pcc* inoculation (Supplementary Figure S1a). Higher levels of CD71+ were also found in BasoE and PolyE populations in reinfected mice at day 3, confirming the capacity of these animals to rapidly restore erythropoiesis. However, all targeted erythroid populations (ProE, BasoE, PolyE) exhibited a significant decrease in the mean fluorescence intensity (MFI) of CD71+ at day 3 post-infection. Additionally, PolyE, OrthoE, and reticulocytes showed significant decreases at day 7 post-infection. BasoE also presented a significant decrease immediately post-infection (day 0) and at day 20 when compared to reinfected, young animals. (Supplementary Figure S2a). As for aged mice, a reduced level of CD71+ cells was already observed at the basal level and became more pronounced 7 days after *Pcc* inoculation (Supplementary Figure S1b). No major differences were observed when evaluating reinfection conditions in aged animals, showing an increase in CD71+ levels before re-inoculation. The enhanced erythropoiesis, detected in relation to younger animals, was consistent with previous results obtained (Supplementary Figure S1b). Upon infection, significant decreases in MFI for CD71+ levels were observed particularly for ProE and BasoE at day 0 and for ProE alone at day 7. The recovered and reinfected group in aged animals did not exhibit significant changes, suggesting a consistent pattern (Supplementary Figure S2b).

### 2.3. Splenic Stress Erythropoiesis in Infected and Reinfected Mice

**Figure 3:**
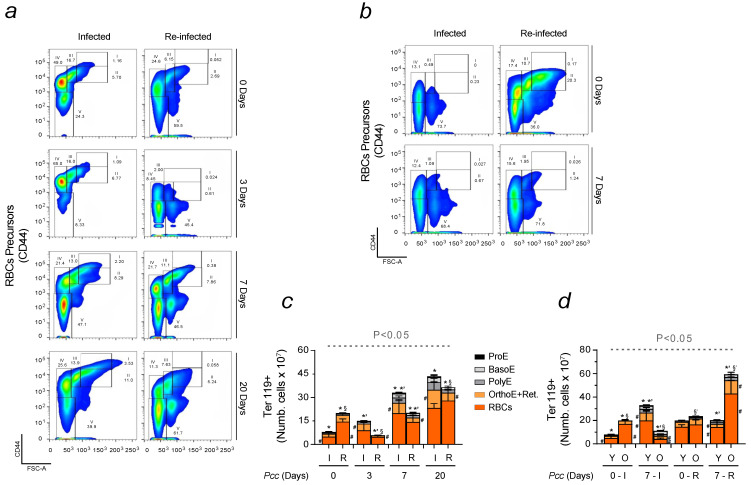
Stress erythropoiesis in *Pcc*-infected and reinfected mice. (**a**) Gating strategy of erythroid populations, isolated from the spleens of infected and reinfected mice on indicated days. Ter119+ cells were analyzed and, based on FSC-A and CD44, five subpopulations were identified: proerythroblasts (I), with high FSC-A and CD44 expression; basophilic erythroblasts (II), with intermediate FSC-A and high CD44 expression; polychromatic erythroblasts (III), with reduced FSC-A and high CD44 expression; orthochromatic erythroblasts and immature reticulocytes (IV), with low FSC-A and high CD44 expression; and mature red blood cells (V), with low FSC-A and CD44 expression. (**b**) Gating strategy to assess the different phases of erythropoiesis in infected and reinfected aged mice. (**c**) Flow cytometry quantification of graphs in (**a**,**d**) or of graphs, as in (**b**), with histograms displaying counts of erythroid subpopulations: proerythroblast (ProE, black), basophilic (BasoE, light grey), polychromatic (PolyE, dark grey), orthochromatic and reticulocyte (OrthoE+Retic, yellow), and mature RBC (RBCs, orange). Data are expressed as mean values ± standard deviations (n = 8–13). Statistically significant differences were calculated via one-way ANOVA. When indicated, significancy refers to the differences that were found when comparing the results of the time course of malaria between infected and reinfected mice. * refers to statistically significant data obtained when comparing non-manipulated mice to infected and reinfected animals at indicated time points. *’ refers to statistically significant data obtained when comparing infected and reinfected mice at specific time points. § refers to statistically significant data obtained when comparing recovered control mice to reinfected animals at indicated time points. §’ refers to statistically significant data obtained when comparing reinfected aged animals at specific time points. # refers to statistically significant data obtained when comparing indicated populations at indicated time points, between infected and reinfected conditions.

Inefficient erythropoiesis, caused by *Plasmodium* infection, is one of the main features of SMA [37,38]. Compensatory mechanisms, referred to as stress erythropoiesis, are engaged to overcome the impaired proliferation of bone marrow. Among those, the most relevant is splenic hematopoiesis, which was, first, assessed through qRT-PCR. The expression of the transcription factor, *GATA-1*, promoting the development of erythroid lineages, was quantified [39,40]. Increased levels were observed in the spleen of infected mice 7 days after *Pcc* inoculation. No significant changes were found in reinfected animals (Supplementary Figure S3a). Our data were consistent with flow cytometry analyses evaluating the erythropoietic capacity of this organ. Despite differences at the basal level and the progressive increase in splenic erythropoiesis, this compensatory mechanism was reduced in reinfected mice (Figure 3a). When assessing extramedullary erythropoiesis in *Pcc*-induced aged mice, their anemic profile presented major differences, already at the basal level (Figure 3b). The counts of Ter119+ cells were quantified in all erythroid populations. When compared to single-infected animals, lower counts of Ter119+ cells were detected in young mice (Figure 3c). As for *Pcc*-induced aged animals, major differences were shown in reinfected conditions (Figure 3d). Stress erythropoiesis is consistent with mice developing splenomegaly. Accordingly, the weight of spleens harvested from infected mice was higher in relation to animals exposed to a second infection. The spleen in reinfected animals was maintained within normal ranges throughout reinfection, which also indicates that this organ was not subjected to the same “stress” experienced by infected mice (Supplementary Figure S3b).

### 2.4. Immune Response Activation in Infected and Reinfected Mice

**Figure 4:**
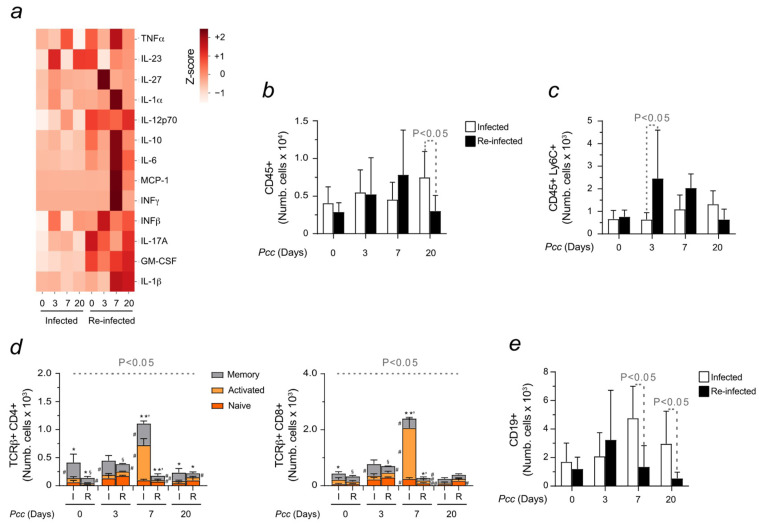
Immune response activation in the blood of infected and reinfected mice. (**a**) Plasma was collected on indicated days from infected and reinfected mice (n = 4 per group). The heatmap represents the normalized (z-score) mean concentration of the 13 cytokines (columns) measured in indicated samples (rows). One-way ANOVA with post-hoc Tukey HSD test was performed for group comparison. Statistically significant differences were found between infected and reinfected animals, being particularly evident on day 7 in reinfected mice. Histogram represents the number of isolated (**b**) peripheral blood leukocytes (CD45+), (**c**) monocytes (CD45+Ly6C+), and (**d**) T cells (CD4+ and CD8+). The activation of T cell subtypes was determined through the expression of CD44 and CD62L, allowing us to identify naïve (CD44−D62L+, orange), effector or activated (CD44+CD62L−, yellow), and memory cells (CD44+CD62L+, grey). (**e**) Circulating B cells (CD19+). Data are expressed as mean values ± standard deviations (n = 7–12). Statistically significant differences were calculated via one-way ANOVA. When indicated, significancy refers to the differences that were found when comparing the results of the time course of malaria between infected and reinfected mice. * refers to statistically significant data obtained when comparing non-manipulated mice to infected and reinfected animals at indicated time points. *’ refers to statistically significant data obtained when comparing infected and reinfected mice at specific time points. § refers to statistically significant data obtained when comparing recovered control mice to reinfected animals at indicated time points. # refers to statistically significant data obtained when comparing indicated populations, at indicated time points, between infected and reinfected conditions.

The cross-talk between erythropoiesis and immunity was investigated in infected and reinfected mice. The activation of the immune response against malaria was first assessed through ELISA. A heatmap was created to measure the release of selected cytokines and chemokines upon *Pcc* inoculation. Different inflammatory profiles were observed, with reinfected mice presenting an enhanced upregulation of MCP-1 and IFN-γ, especially at day 7, in relation to infected animals. Although less pronounced, an increased release of pro-inflammatory cytokines was also found in infected mice during malaria (Figure 4a). While the results obtained were consistent with the significant changes in immune activation described with the development of this disease [18], no major differences were observed between the conditions tested in relation to the total number of leukocytes in circulation. Only on day 20, changes in the counts of CD45+ cells were observed. Higher numbers were found in infected but not reinfected animals, consistent with a more rapid recovery of these latter (Figure 4b). In agreement, reinfected animals showed an increased monocytic population, as assessed through the abundance of Ly6C, within CD45+ cells (Figure 4c). As for adaptive immunity, a significant increase in the number and activation of both CD4+ and CD8+ T cells was shown in infected mice at the peak of parasitemia (Figure 4d), which was consistent with the required time for the engagement of adaptive immunity [41]. The activation of those cells was assessed through the levels of CD44 and CD62 markers. Regarding B cells, the increased counts of CD19+ population in infected mice at later time points, day 7 and day 20, contrasted with the lower numbers found in reinfected animals (Figure 4e). Representative plots confirmed the results described (Supplementary Figure S4).

**Figure 5:**
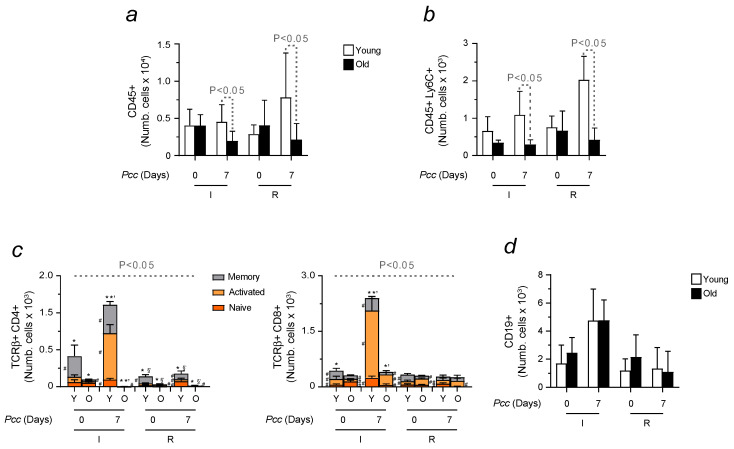
Immune response activation in the blood of infected and reinfected young vs. aged mice. Histogram represents the number of isolated (**a**) peripheral blood leukocytes (CD45+), (**b**) monocytes (CD45+Ly6C+), and (**c**) T cells (CD4+ and CD8+). The activation of T cell subtypes was determined through the expression of CD44 and CD62L, allowing us to identify naïve (CD44–CD62L+, orange), effector or activated (CD44+CD62L–, yellow), and memory cells (CD44+CD62L+, grey). (**d**) Circulating B cells (CD19+). Data are expressed as mean values ± standard deviations (n = 7–10). Statistically significant differences were calculated via one-way ANOVA. When indicated, significancy refers to the differences that were found when comparing the results of the time course of malaria between infected and reinfected mice. * refers to statistically significant data obtained when comparing non-manipulated mice to infected and reinfected animals at indicated time points. *’ refers to statistically significant data obtained when comparing infected and reinfected mice at specific time points. §’ refers to statistically significant data obtained when comparing reinfected animals at specific time points. # refers to statistically significant data obtained when comparing indicated populations, at indicated time points, between infected and reinfected conditions.

When evaluating the immune response in aged animals developing malaria, we observed a reduced capacity to resolve the infection. In relation to younger animals, they showed lower counts of CD45+ cells and a reduced monocytic population (Figure 5a,b). Their inability to activate the adaptive immunity was also observed upon *Pcc* reinfection (Figure 5c). No difference in the number of B cells was found among tested conditions (Figure 5d). Representative plots confirmed the results obtained (Supplementary Figure S5).

**Figure 6:**
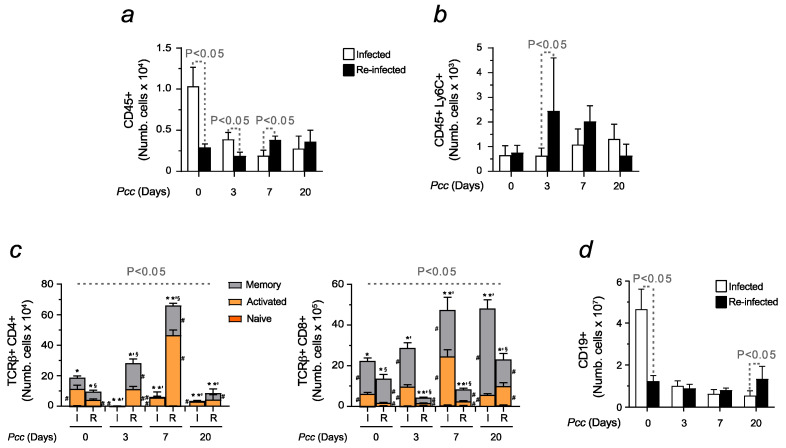
Immune response activation in the bone marrow of infected and reinfected mice. Histogram represents the number of isolated (**a**) bone marrow leukocytes (CD45+), (**b**) monocytes (CD45+Ly6C+), and (**c**) T cells (CD4+ and CD8+). The activation of T cell subtypes was determined through the expression of CD44 and CD62L, allowing us to identify naïve (CD44−CD62L+, orange), effector or activated (CD44+CD62L−, yellow), and memory cells (CD44+CD62L+, grey). (**d**) B cells (CD19+). Data are expressed as mean values ± standard deviations (n = 7–12). Statistically significant differences were calculated via one-way ANOVA and are indicated accordingly. When indicated, significancy refers to the differences that were found when comparing the results of the time course of malaria between infected and reinfected mice. * refers to statistically significant data obtained when comparing non-manipulated mice to infected and reinfected animals at indicated time points. *’ refers to statistically significant data obtained when comparing infected and reinfected mice at specific time points. § refers to statistically significant data obtained when comparing recovered control mice to reinfected animals at indicated time points. # refers to statistically significant data obtained when comparing indicated populations, at indicated time points, between infected and reinfected conditions.

When evaluating the immune response in organs responsible for RBC production, such as the bone marrow, we observed a dramatic decrease in the number of leukocytes during the infection whereas the counts of CD45+ cells were maintained almost unchanged in reinfected animals (Figure 6a). The same trend was displayed by the monocytic population (Figure 6b). As for adaptive immunity, opposite patterns were found when referring to CD4+ and CD8+ T cell activation. While the former was enhanced in reinfected mice, higher counts, and the activation of CD8+ T cells, were shown in animals exposed to a single infection (Figure 6c). When assessing B cells, major differences were found at basal levels, with lower numbers upon reinfection. Opposite trends were detected only 20 days after *Pcc* inoculation (Figure 6d). Representative plots confirmed the results described (Supplementary Figure S6).

**Figure 7:**
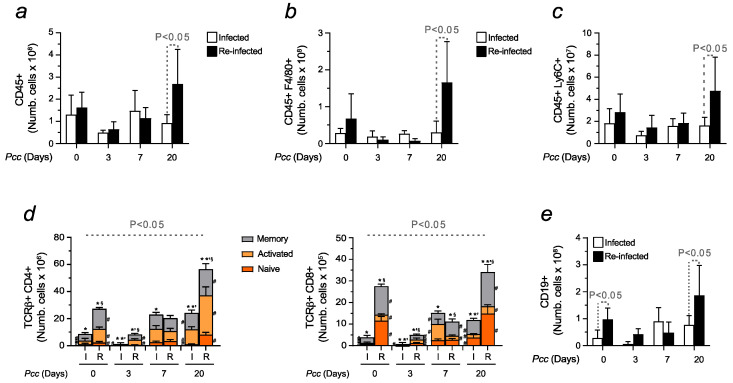
Immune response activation in the spleens of infected and reinfected mice. Histogram represents the number of isolated (**a**) bone marrow leukocytes (CD45+); (**b**) erythrophagocytes (CD45+F4/80+); (**c**) monocytes (CD45+Ly6C+); (**d**) isolated T cells (CD4+ and CD8+), the activation of which was determined through the expression of CD44 and CD62L, allowing us to identify naïve (CD44−CD62L+, orange), effector or activated (CD44+CD62L−, yellow), and memory cells (CD44+CD62L+, grey); and (**e**) B cells (CD19+). Data are expressed as mean values ± standard deviations (n = 7–12). Statistically significant differences were calculated via one-way ANOVA. When indicated, significancy refers to the differences that were found when comparing the results of the time course of malaria between infected and reinfected mice. * refers to statistically significant data obtained when comparing non-manipulated mice to infected and reinfected animals at indicated time points. *’ refers to statistically significant data obtained when comparing infected and reinfected mice at specific time points. § refers to statistically significant data obtained when comparing recovered control mice to reinfected animals at indicated time points. # refers to statistically significant data obtained when comparing indicated populations, at indicated time points, between infected and reinfected conditions.

When evaluating the spleen, no differences were observed in the levels of CD45+, except at the late stages of *Pcc*-induction, when reinfected animals presented higher counts (Figure 7a). Given the important role of the spleen in Fe recycling [42,43], changes in the number of erythrophagocytes were assessed among tested conditions, quantifying splenic red pulp macrophages through the F4/80 marker. Measured within the leukocyte population, a significant increase in the prevalence of F4/80+ cells was detected in reinfected mice at day 20 (Figure 7b), which was consistent with trends also showed by splenic monocytes (Figure 7c). As for adaptive immunity, reinfected mice presented higher counts of CD4+ and CD8+ T cells, particularly evident at the basal level and at day 20 (Figure 7d). The same tendency was also shown to that concerning B cells (Figure 7e). Representative plots confirmed the results obtained (Supplementary Figure S7).

### 2.5. Erythropoietic Response in Infected Immunocompromised Mice

**Figure 8:**
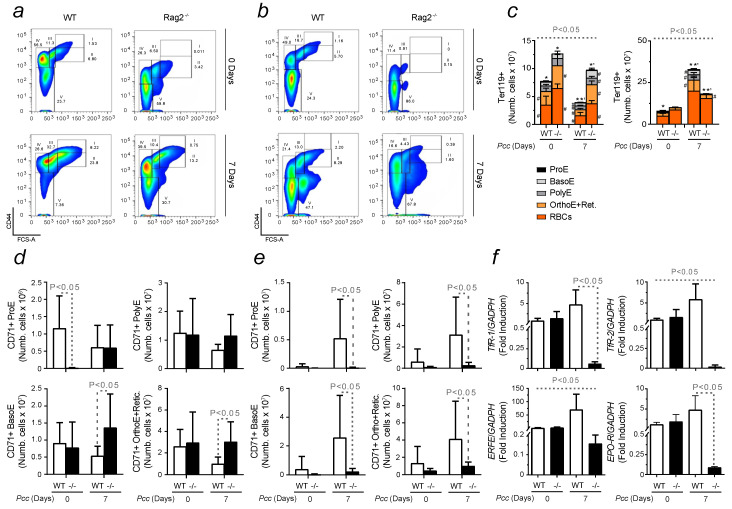
Erythroid lineage evaluation in infected wild-type vs. Rag2^−/−^ mice. (**a**) Gating strategy for erythroid populations, in (**a**) bone marrow and (**b**) spleen, of infected wild-type and Rag2^−/−^ mice at indicated days. Ter119+ cells were analyzed and, based on FSC-A and CD44, five subpopulations were identified. Proerythroblasts (I) showed higher expression of CD44 and FSC-A; basophilic erythroblasts (II), intermediate FSC-A and high CD44 expression; polychromatic erythroblasts (III), reduced FSC-A and high CD44 expression; orthochromatic erythroblasts and immature reticulocytes (IV), low FSC-A and high CD44 expression; and mature red blood cells (V), low FSC-A and CD44 expression. Flow cytometry quantification of erythroid cells, collected from (**c**) bone marrow and spleen samples from wild-type (WT) and Rag2^−/−^ (−/−) mice, at day 0 and 7 post-infection. Histograms display the number of cells of each erythroid subpopulation: proerythroblast (ProE, black), basophilic (BasoE, light grey), polychromatic (PolyE, dark grey), orthochromatic and reticulocyte (OrthoE+Retic, yellow) and mature RBC (RBCs, orange). Quantification, in (**d**) bone marrow and (**e**) spleen, of total proerythroblast (ProE), basophilic (BasoE), polychromatic (PolyE), orthochromatic, and reticulocyte (OrthoE+Retic) cells expressing the TfR1/CD71 marker. (**f**) Transferrin receptor 1 (TfR-1), 2 (TfR-2), Erythroferrone (ERFE) and erythropoietin receptor (EPO-R) mRNA expression, quantified via qRT-PCR, in bone marrow samples from infected and reinfected wild-type and Rag2^−/−^ mice. Data are presented as mean values ± standard deviations (n = 8-13). Statistically significant differences were calculated via one-way ANOVA. When indicated, significancy refers to the differences that were found when comparing the results of the time course of malaria between infected and reinfected mice. * refers to statistically significant data obtained when comparing non-manipulated wild-type mice to infected animals from the same genotype as well as to Rag2^−/−^ at indicated time points. *’ refers to statistically significant data obtained when comparing wild-type and Rag2^−/−^ infected mice at day 7 post-infection. # refers to statistically significant data obtained when comparing indicated populations, at indicated time points, between infected and reinfected conditions.

A loss-of-function approach was used to assess the contribution of the immune system to the development of SMA and to evaluate the interplay between immunity and erythropoiesis. The erythropoietic capacity of immunocompromised Rag 2^−/−^ mice, lacking T and B cells, was investigated upon *Pcc* inoculation. Rag 2^−/−^ animals were exposed only to a single infection as their inability to trigger an appropriate immune response would lead those mice to succumb to malaria-induced hyperparasitemia during the recovery period [44]. In agreement with literature findings [45], our data demonstrated that adaptive immunity plays an important role in shaping erythropoiesis. Representative graphs and correspondent plots, illustrating different erythroid populations, are shown for the bone marrow (Figure 8a) and spleen (Figure 8b). Major differences were observed between wild-type and Rag 2^−/−^ animals already at the basal level. A higher number of Ter119+ cells was found in the bone marrow of Rag 2^−/−^ mice, in relation to genotype controls, at the peak of the infection, day 7. These data demonstrated that erythropoiesis was enhanced in the absence of T and B cells. Although in the spleen, a comparable level of mature RBCs was detected at basal levels, the number of erythroid precursors in this organ was higher in wild-type mice upon infection (Figure 8c). This observation was consistent with a decreased requirement for extramedullary erythropoiesis in Rag 2^−/−^ animals upon infection. The rapid expansion of erythroid populations led to an increased number of erythroid cells expressing TfR1/CD71 in Rag 2^−/−^ mice, indicating an enhanced requirement for Fe uptake [46]. Higher levels of TfR1/CD71 were found in Rag 2^−/−^ in the bone marrow (Figure 8d). Further validating these results, we observed significantly higher levels of median fluorescence of this parameter in the different precursors of the bone marrow upon the infection of immunocompromised animals. The opposite trend was found in the spleens of these animals (Supplementary Figure S8). The influence of adaptive immunity in modulating RBC production was also confirmed by quantifying the expression of receptors for iron uptake, like *TfR1* and *TfR2*, in bone marrow samples through qRT-PCR. The significant increase in *TfR1* and *TfR2* in wild-type mice during infection was consistent with the need to increase the production of RBCs to cope with *Pcc*-induced hemolysis. Conversely, the tendency shown by Rag 2^−/−^ mice suggested the interference of non-erythroid cells in the analysis, considering the opposite results obtained in relation to iron intracellular uptake. Nevertheless, the improved erythropoietic performance of Rag2^−/−^ mice was further supported by the expression of erythroferrone (*ERFE*), which was higher in wild-type mice during infection. Its function to inhibit hepcidin activity, releasing the restriction imposed by that hormone on Fe availability, confirmed the need of wild-type mice to increase erythropoiesis upon infection. This was also indicated by the higher expression of erythropoietin receptor (*EPO-R*) in bone marrow samples from those animals when compared to Rag 2^−/−^ mice (Figure 8e). Considering the different cell population contained in those extracts, further analyses are required to assess each contribution to uptake Fe and improve erythropoiesis.

Overall, these data revealed that the immune system regulates the production of RBCs during infection, shaping erythropoiesis to possibly prevent Fe cytotoxicity and tissue damage.

## 3. Discussion

The cross-talk between immunity and erythropoiesis becomes highly relevant in the context of hemolytic diseases like malaria. Erythrocytes have been found capable to exert immunomodulatory functions, influencing host–pathogen interaction and, consequently, the activation of the immune response elicited against parasite invasion [47]. Intrinsic pro-inflammatory properties have been attributed to non-hemoglobin-bound heme when released into circulation upon hemolysis [17,48], and endogenous strategies have been evolutionarily developed to prevent the tissue damage it induces [49].

This study aimed at providing a better understanding on the development of SMA and the contribution of immune cells to its progression. The influence of the immune system in shaping RBC production is potentially due to defense strategies activated to protect host tissues from the stress imposed by Fe cytotoxicity, as indicated by our study.

Ineffective erythropoiesis is one of the main features of SMA, a complication that significantly contributes to increase malaria mortality, especially in children and primigravidae [50]. A higher incidence of SMA was reported upon multiple malaria exposures. To mimic common occurrences in endemic areas, previously infected C57BL/6 mice were newly exposed to malaria after being left to recover for a period of 2 months. Comparative analyses were performed to investigate the influence of immune system activation on erythropoiesis in infected and reinfected conditions. A strain of *Plasmodium* causing a high degree of hemolysis was used to conduct our experiments [17]. A peak in parasitemia was consistently observed 7 days after infection, a time point used for comparisons corresponding to the maximum hemolysis [51]. Malaria is a Fe-deficient disease, characterized by high levels of hypoxia, that is known to impact RBC production. Erythropoiesis is regulated by EPO, a hormone produced by the kidney and acting on the bone marrow [30,31]. Changes in EPO signaling were assessed in infected and reinfected mice (Figure 1). While both showed the ability to activate the EPO pathway, major differences were observed in terms of hormone production and release into circulation. Reinfected mice performed better as a higher concentration of EPO was measured in blood despite reduced RBC counts in the first phase of the infection (Figure 2). It is known that the higher the oxygenation is, the lower the damage caused by impaired tissue function will be. However, it is worth highlighting that this advantage was not due to parasite burden as at day 7, no significant differences were found between infected and reinfected mice. To mimic malaria affecting the elderly, older mice of approximately one year in age were infected to evaluate potential differences in erythropoiesis and its immune regulation caused by advancing age. Despite a similar response to the single infection, older mice were more sensitive to malaria re-exposure (Figure 2). When compared to younger animals, an increased reduction in the number of RBCs sensitized aged mice to develop greater SMA and succumb to reinfection 7 days after *Pcc* inoculation. The limiting factor for RBC production is Fe availability [29,32]. When assessing Fe uptake through the abundance of TfR1/CD71 in erythroid populations, major changes were observed between infection and reinfection conditions. While, in single-infected animals, there were fewer erythroid precursors positive for CD71+, those cells presented a higher MFI, suggesting an enhanced Fe uptake and requirement when compared to reinfected animals. A different behavior was found when analyzing young and old mice. In response to malaria, the former enhanced not only the number of cells positive to CD71 but also the expression of this marker on erythroid precursors. This translated into a lower Fe uptake in old mice, which was consistent with an increased disease severity, presumably caused by the high levels of Fe deficiency. Inefficient erythropoiesis is associated with the activation of compensatory mechanisms, promoting erythroid proliferation. Extramedullary RBC production mainly occurs in the spleen. Possibly, this constitutes an energy-saving strategy as the organ devoted to Fe recycling also acts as the main producer of erythrocytes. An enhanced splenic erythropoiesis was observed upon infection. The activation of stress erythropoiesis in reinfected mice was lower, confirming that these animals did not require the spleen to generate significant numbers of RBCs. This notion was further supported by the lower weight of this organ in mice newly exposed to *Plasmodium*. Reinfected animals did not present the typical signs of splenomegaly, which is one of the features of malaria (Figure 3). Although not to the same extent, there are other organs with the ability to compensate the inefficient erythropoiesis of the bone marrow. These include the liver, and since this organ is responsible for Fe storage [38], it strengthens the hypothesis that during malaria, tissue-specific energy-sparing strategies are engaged to overcome Fe deficiency.

The differences In erythropoiesis observed under the conditions tested encouraged us to assess the impact of immune system activation on this process upon single or multiple malaria exposures. Innate immune cells are activated by “inducers” that are also released, by *Plasmodium*, as heme [52] or its derived pigment hemozoin [53]. The damage induced by the hemolytic nature of malaria led us to compare pro-inflammatory markers released into circulation (Figure 4). Their concentration was found to be higher in reinfected mice, being already at the basal level. However, major differences were observed during the evolution of malaria, with a pronounced pro-inflammatory cytokine release occurring upon reinfection. This robust immune response was consistent with increased innate immune cell populations in the circulation in reinfected mice. Significant changes were also observed in relation to the adaptive immunity as infected mice increased T and B cell activation in relation to reinfected animals, especially at the peak of parasitemia. When the same analyses were conducted in older animals, their decreased capacity to elicit an immune response was significantly detected. A reduced count was found in all evaluated immune cell subsets (Figure 5), which was consistent with their vulnerability to the infection that led mice to succumb to a second exposure [35]. The engagement of the immune response was also evaluated in organs producing RBCs, with the aim to verify the existence of a potential immune regulation of erythropoiesis. In the bone marrow, opposite trends of CD4+ and CD8+ T cell activation were observed when comparing infected and reinfected mice (Figure 6). While activated CD4+ T cells prevailed in reinfected mice, the memory phenotype of CD8+ T cells characterized animals exposed to a single infection. Whether this behavior may underlie the ability of reinfected mice to adapt to stimuli, developing a “trained immunity” that persists upon re-exposures is likely the case [54,55]. Major changes in B cell counts were also observed in the bone marrow. The decrease in the number of B cells at the basal level in reinfected mice contrasted with their increase during the last phases of malaria when compared to single-infected animals. It is important to highlight that our data aligned with published studies showing a pronounced B cell expansion upon reinfection [56,57,58]. Similar trends were also found in the spleen concerning the profile of activated CD4+ T cells, which was higher in reinfected mice (Figure 7). Those results were consistent with studies conducted in Uganda that reported an increased CD4+ T cell differentiation in children repetitively exposed to malaria. Multiple infections were also found to induce transcriptional changes in CD8+ T cell activation [59] as described in Western Kenyan cohorts [60]. In our experiments, an increased memory phenotype characterized the response of splenic CD8+ T cells to malaria. Enhanced B cell expansion in the spleen was detected upon reinfection, in agreement with the generation of a long-term trained memory [61]. Malaria is a multiorgan disease. Hence, it requires a robust immune activation to resolve the infection and clear *Plasmodium* parasites [18]. However, the expansion of immune cells is a Fe-dependent process. If, from one side, this metal is necessary for RBC production, from the other, it allows the immune system to better cope with the infection [62]. Hence, consistent with the role of the spleen in Fe recycling, an increased population of hemophagocytic macrophages was detected in reinfected mice (Figure 7).

To further prove the influence of the immune system on RBC production, a loss-of-function approach was used. Immunocompromised mice, lacking adaptive immunity, were infected with *Plasmodium* (Figure 8). The response of Rag2^−/−^ mice was compared to that of wild-type mice. Our data showed that immunocompromised animals activated a more efficient bone marrow erythropoiesis, being already at the basal level. Although this effect was maintained during the infection, the absence of T and B cells led those mice to succumb to malaria within the recovery phase [63]. The enhanced erythropoiesis was not sufficient to prevent the lethality caused by hyperparasitemia. However, Rag 2^−/−^ animals presented a reduced extramedullary erythropoiesis in response to malaria and compared to the wild type, demonstrating that adaptive immunity exerts an important role in shaping RBC production. When compared to controls, a higher expression of TfR1/CD71 was found in immunocompromised mice, particularly in the bone marrow. This was possibly due to the need to preserve tissue function, providing oxygen to cells as long as possible before succumbing to the infection. The higher expression of genes related to Fe uptake by erythroid cells, namely *TfR1* and *TfR2*, in wild-type animals confirmed that mice deprived of adaptive immunity do not need much Fe to show an improved erythropoiesis during malaria. However, multiple cell populations are found in the bone marrow, which could explain the opposite results obtained, when assessing gene expression. In agreement with the improved erythropoiesis observed in Rag 2^−/−^ mice, lower and almost undetectable levels of *ERFE* were also shown, further proving the existence of a tight regulation between the immune system and Fe metabolism. The result of an improved RBC production upon infection by Rag 2^−/−^ mice was also supported by the reduced expression of *EPO-R* expression in bone marrow samples.

Our data demonstrate the existence of complex immune-regulatory mechanisms of erythropoiesis, which could be therapeutically explored. A combination of treatments targeting the immune system and parasite proliferation could be tested to improve anemia, paying attention to not impair the response and severity of *Plasmodium* infection.

## 4. Materials and Methods

### 4.1. Mice

Wild-type C57BL/6 mice, females and males, aged 8–12 weeks (referred to as young) or 52–60 weeks (referred to as old), were used for this study. Mice were purchased from the animal facility production of the Champalimaud Foundation, Alges, Portugal. Non-infected animals were used as experimental controls. Rag 2^−/−^ C57BL/6 mice were purchased from the animal facility production of the Gulbenkian Institute of Science, Oeiras, Portugal.

All mice were bred and maintained under specific pathogen-free (SPF) conditions. Animal care and experimental procedures were conducted in accordance with Portuguese guidelines and regulations, following the approval received by the respective local (Champalimaud Foundation, where colonies are maintained) and governmental committees (DGAV).

### 4.2. Malaria Induction and Parasitemia Count

Malaria was induced by infecting experimental mice with *Pcc* AS strain. *Pcc*-infected RBCs were collected from a donor mouse. The infection was then performed intraperitoneally (i.p.), with an injection of approximately 10^6^ *Pcc*-infected RBCs, in a volume of 200 μL. The reinfection model was reproduced by allowing mice to recover for 2 months, while monitoring for complete parasite clearance, before re-exposing animals to the same infection modality.

Parasitemia was monitored daily, performing a Giemsa-stain of blood smears (Sigma, Ref. No. 48900; Darmstadt, Germany).

### 4.3. Single-Cell Suspension for Flow Cytometry Analysis of Erythroid Lineage

BM, spleen, and liver samples were collected, after perfusing mice with cold PBS 1×, as follows. Both femur and tibia were cut at both ends and BM was flushed with a 26-gauge needle (Braun, Ref. No. 4657683; Melsungen, Germany) attached to 1mL syringe (Terumo, Ref. No SS+01H1; Tokyo, Japan) filled with ice-cold FACS buffer. The spleen was smashed in a 6mm petri dish. The sample was homogenized by using a 100 µm mesh (LINKER Industrie-Technik, Ref. No. 11774539; Kassel, Germany) in FACS buffer containing PBS 1× with 2% heat inactivated FBS (Gibco, Ref. No. 10270-106; Waltham, MA, USA). The single-cell suspension was then transferred to a 2 mL Eppendorf tube and resuspended in a total of 2 mL of FACS buffer. The liver was separated into different lobules, removed, and smashed with a 20 mL syringe (TERUMO, Ref. No. MDSS20ESE; Tokyo, Japan) on a 100 µm strainer (Falcon, Ref. No. 352360; Schiphol, The Netherlands). The homogenized pellet was then centrifuged in a 50 mL Falcon tube containing 35% Percoll (GE Healthcare, Ref. No.17-0891-01; Darmstadt, Germany). The supernatants were collected and washed with PBS 1×. Finally, cells were suspended in FACS buffer for cell counting and for further analyses.

### 4.4. Single-Cell Suspension for Flow Cytometry Analysis of Leukocytes 

After plating cells from spleen and liver suspension for erythropoietic analyses, the samples were processed as follows. Cell suspensions were centrifuged for 2 min at 2000 rpm at room temperature. The supernatant was discarded and the pellet resuspended in RBC lysis buffer (1×), for 10 min at room temperature. The samples were then centrifuged for 5 min, at 1500 rpm, and washed twice with FACS buffer. After we discarded the supernatant, splenic cells were resuspended in 2 mL of FACS buffer while liver cells were resuspended in 450 µL of FACS buffer (150 µL/antibody mix).

As for the blood, samples were centrifuged for 5 min at 1600× *g* at room temperature. Cells were resuspended in RBC lysis buffer (2×) (BioLegend, Ref. No. 420301; Amsterdam, The Netherlands) in a 1:1 proportion (pellet volume vs. buffer volume) and left for 10 min at room temperature. The samples were centrifuged for 5 min at 1500 rpm, and the supernatant was discarded. The process of RBC lysis was repeated twice. Cells were washed twice with FACS buffer and resuspended in 450 µL of FACS buffer (150 µL/antibody mix).

Please note that BM samples did not require further processing.

### 4.5. Flow Cytometry

The total number of infiltrated immune cells was measured through flow cytometry using a known concentration of reference 10 μm latex-bead suspension (Polysciences Europe GmbH, Ref. No CC10N-10; Hirschberg an der Bergstrasse, Germany), co-acquired with a pre-established volume of cellular suspension. Dead cells were excluded using Propidium Iodide. Singlets were gated among live cells based on size and granularity. Cells were stained with Fc block (anti-CD16/CD32; BD Pharmingen™-Ref. No 553141; Madrid, Spain) to prevent non-specific binding for 20 min at room temperature. Cells were then washed in PBS supplemented with 2% heat-inactivated FCS and stained as follows. 

The expression level of CD44, as a function of FSC in all Ter119 positive cells, allowed us to obtain highly purified populations of erythroblasts such as proerythroblasts, basophilic erythroblasts, polychromatic erythroblasts, orthochromatic erythroblasts, and immature reticulocytes [64]. In each of these populations, we also measured the levels of CD71 used to quantify the levels of TfR1 [59]. Anti-TCR-β and anti-CD19 allowed us to identify T cells and B cells, respectively. Within the T cell family, it was possible to identify cytotoxic, helper, and double-positive T cell subpopulations by using anti-CD8 and anti-CD4, respectively. Anti-CD44 and anti-CD62L allowed determining the levels of activation of CD4^+^ and CD8^+^ cells. Anti-CD45 allowed identifying leukocytes, from which macrophages were distinguished via the expression of F4/80. Monocytes and neutrophils were identified through the combination of anti-Ly6C and anti-Ly6G. It was possible to further confirm the detection of neutrophils by using anti-CD11b.

Antibodies were purchased from BioLegend (PE/Cy7 anti-mouse/human CD11b Antibody, Ref. No. 101216; Brilliant Violet 421 anti-mouse CD45 Antibody, Ref. No. 103134; APC anti-mouse Ter119 Antibody, Ref No. 116212; PE anti-mouse/human CD44 Antibody, Ref No. 103007; PE anti-mouse TCR-β chain Antibody, Ref No. 109208; PE/Cy7 anti-mouse CD62L Antibody, Ref. No. 104417; Brilliant Violet 510 anti-mouse CD19 Antibody, Ref. No. 115545; APC anti-mouse/human CD44 Antibody, Ref. No.103012; APC/Cy7 anti-CD8, Ref. No. 100714; APC/Cy7 anti-mouse CD45 Antibody, Ref. No. 103115; BV510 anti-mouse F4/80 Antibody, Ref. No. 123135; FITC anti-mouse CD71 Antibody, Ref. No. 113805; FITC anti-mouse Ly6C Antibody, Ref. No. 128006; APC anti-mouse Ly6G Antibody, Ref. No. 127613; Brilliant Violet 510 anti-mouse TCR-β Antibody, Ref. No. 109233; Amsterdam, The Netherlands) and BD Biosciences (Pacific Blue anti-mouse CD4 Antibody, Ref. No. 558107; PE Rat Anti-Mouse Ly6G and Ly6C, Ref. No. 553128; and Pacific Blue anti-mouse CD4 Antibody, Ref. No. 558107; Madrid, Spain). Analyses were performed by using BD FACSCanto II Flow Cytometer and the LEGENDplex data analysis software (LEGENDplex v8.0, BioLegend; Amsterdam, The Netherlands).

### 4.6. Enzyme-Linked Immunosorbent Assay (ELISA)

The Legend Max™ Mouse EPO ELISA Kit (Biolegend, Ref. No. 442707; Amsterdam, The Netherlands) was used for the quantitative determination of EPO plasma levels. Absorbance values were recorded in a Synergy HT microplate reader (Biotek; Marshall Scientific, Cambridge, UK; London, UK). Cytokine concentrations were determined using a LEGENDplex mouse inflammation panel (BioLegend, Ref. No. 740446; Amsterdam, The Netherlands) according to the manufacturer’s instructions.

### 4.7. Gene Expression Analysis via Quantitative Real-Time PCR (qRT-PCR)

Kidney and bone marrow samples were collected and processed using tripleXtractor (GRISP, Ref. No GB23.0100; Porto, Portugal) and Phenol:Chloroform:Isoamyl Alcohol 25:24:1 (Sigma-Aldrich, Ref. No. P3803-100ML; Darmstadt, Germany). After RNA extraction, performed by using NucleoSpin^®^ RNA kit (Macherey-Nagel, Ref. No 740955.250; Düren, Germany) according to the manufacturer’s instructions, sample quality and quantity were determined with the NanoDrop 2000c spectrophotometer (Thermo Scientific; Waltham, MA, USA). Total RNA was reverse transcribed into cDNA through the SuperScript III First-Strand Synthesis System for quantitative real-time PCR (RT-qPCR) (Invitrogen, Ref. No. 18080051; Waltham, MA, USA). Purified RNA samples were denatured via heat shock at 90 °C, for 2 min, and then chilled on ice, in agreement with the manufacturer’s instruction. RT-qPCR was performed using the ABI QuantStudio-384 (Applied Biosystems; Waltham, MA, USA). Transcript number was calculated from the threshold cycle (Ct) of each gene with a 2^−ΔΔCT^ method (relative number), normalized to GADPH and expressed as fold induction of animals used as controls. PCR primers included the following—*EPO* Fwd: 5′-GCCCTGCTAGCCAATTCC-3′; *EPO* Rev: 5′-GGCGACATCAATTCCTTCTG-3′; *EPOR* Fwd: 5′-GAAACGACCACTGCTAAGGCA-3′; *EPOR* Rev: 5′-GGCAGACAGCTTAAGGCTCCT-3′; *ERFE* Fwd: 5′-ATGGGGCTGGAGAACAGC-3′; *ERFE* Rev: 5′-TGGCATTGTCCAAGAAGACA-3′; *GADPH* Fwd: 5′-ACCACAGTCCATGCCATCAC-3′; *GADPH* Rev: 5′-CACCACCCTGTTGCTGTAGCC-3′; *HIF-2α* Fwd: 5′-GAAACGACCACTGCTAAGGCA-3′; and *HIF-2α* Rev: 5′-GGCAGACAGCTTAAGGCTCCT-3′.

### 4.8. Software and Statistical Analysis

All flow cytometry data were analyzed by using FlowJo software (version 10.0.7, Tree Star Inc., Ashland, OR, USA).

Statistically significant differences were assessed among the conditions tested via one-way ANOVA, applying Tukey’s Honestly Significant Difference (HSD) test. The means of two groups were compared by using the unpaired *t*-test. Pairwise comparisons among groups were conducted by applying the two-way ANOVA, followed by Sidak’s multiple comparison test. Data were expressed as mean values ± standard deviations. Statistical analysis was performed using GraphPad Prism (GraphPad Software, version 9.0; Boston, MA, USA), indicating significancy as *p* < 0.05.

Cytokine concentrations were determined using the LEGENDplex data analysis software (LEGENDplex v8.0, BioLegend; Amsterdam, The Netherlands). After preprocessing, the data obtained from cytokine concentrations were analyzed using Python 3 in a Jupyter notebook (version 6.4.12). Average concentrations for each cytokine were calculated on different days. For statistical analyses, the one-way ANOVA was applied to explore significant differences, and it was followed by Tukey’s HSD test for post hoc comparisons. Data were visualized by generating a heatmap with hierarchical clustering, allowing us to identify concentration patterns over different time points. As Python libraries, we used pandas, seaborn, matplotlib, numpy, scipy, and statsmodels, with a significance level set at 0.05, to ensure the robustness of the results.

## 5. Conclusions

Our study compared the response to single and multiple malaria exposures in terms of erythropoiesis and immune cell activation. A cross-talk between these processes was observed, along with a certain degree of unresponsiveness of reinfected mice to higher levels of EPO released into circulation. Major differences were found, especially regarding the greater need of infected mice to trigger compensatory mechanisms like splenic erythropoiesis to rapidly overcome Fe deficiency. The limited induction of stress erythropoiesis in reinfected mice suggested that the production of RBCs in the bone marrow is sufficient to cope with malaria. Comparisons between younger and older animals were also established, with these latter suffering major impairments that led to lethal outcomes. The impact of the immune system on erythropoiesis was finally confirmed in immunocompromised mice, showing an enhanced RBC production in the absence of T and B cells. Hence, the ability of the immune system to shape erythropoiesis provides additional avenues to explore novel approaches against infection-driven anemia. The insights offered by our study on the regulation of SMA and the impact of the immunity on its evolution indicate immune cells as potential targets for therapeutic modulation so as to restore the erythropoietic capacity in the context of infectious diseases.

## Data Availability

The data that support the findings of this study are available from the corresponding author upon reasonable request.

## Supplementary Materials

The following supporting information can be downloaded at: https://www.mdpi.com/article/10.3390/ijms25116153/s1.
