# Supplementary material for: Infection vs. Reinfection: The Immunomodulation of Erythropoiesis
Source: Int J Mol Sci. 2024 Jun 3;25(11):6153. doi: 10.3390/ijms25116153 (PMC11172545; doi:10.3390/ijms25116153)
Supplement: Supplementary file 1 [file ijms-25-06153-s001.zip › ijms-3020176-supplementary.pdf]

Suppl. Fig. S1

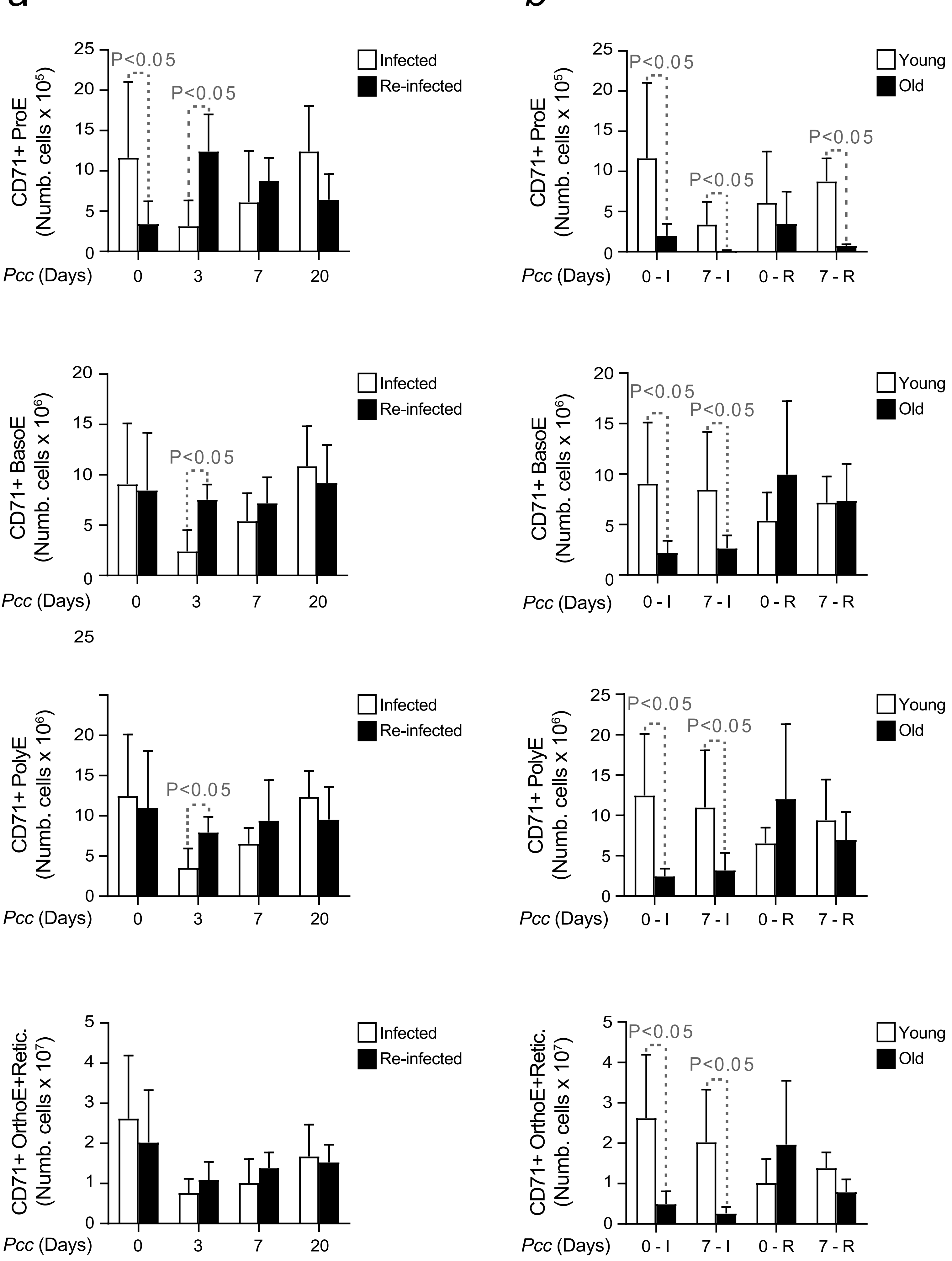
a
b
CD71+ ProE (Numb. cells x 10^5)
CD71+ BasoE (Numb. cells x 10^6)
CD71+ PolyE (Numb. cells x 10^6)
CD71+ OrthoE+Retic. (Numb. cells x 10^7)
Infected
Re-infected
Young
Old
P<0.05
Pcc (Days)
0
3
7
20
0 - I
7 - I
0 - R
7 - R

Suppl. Fig. S2

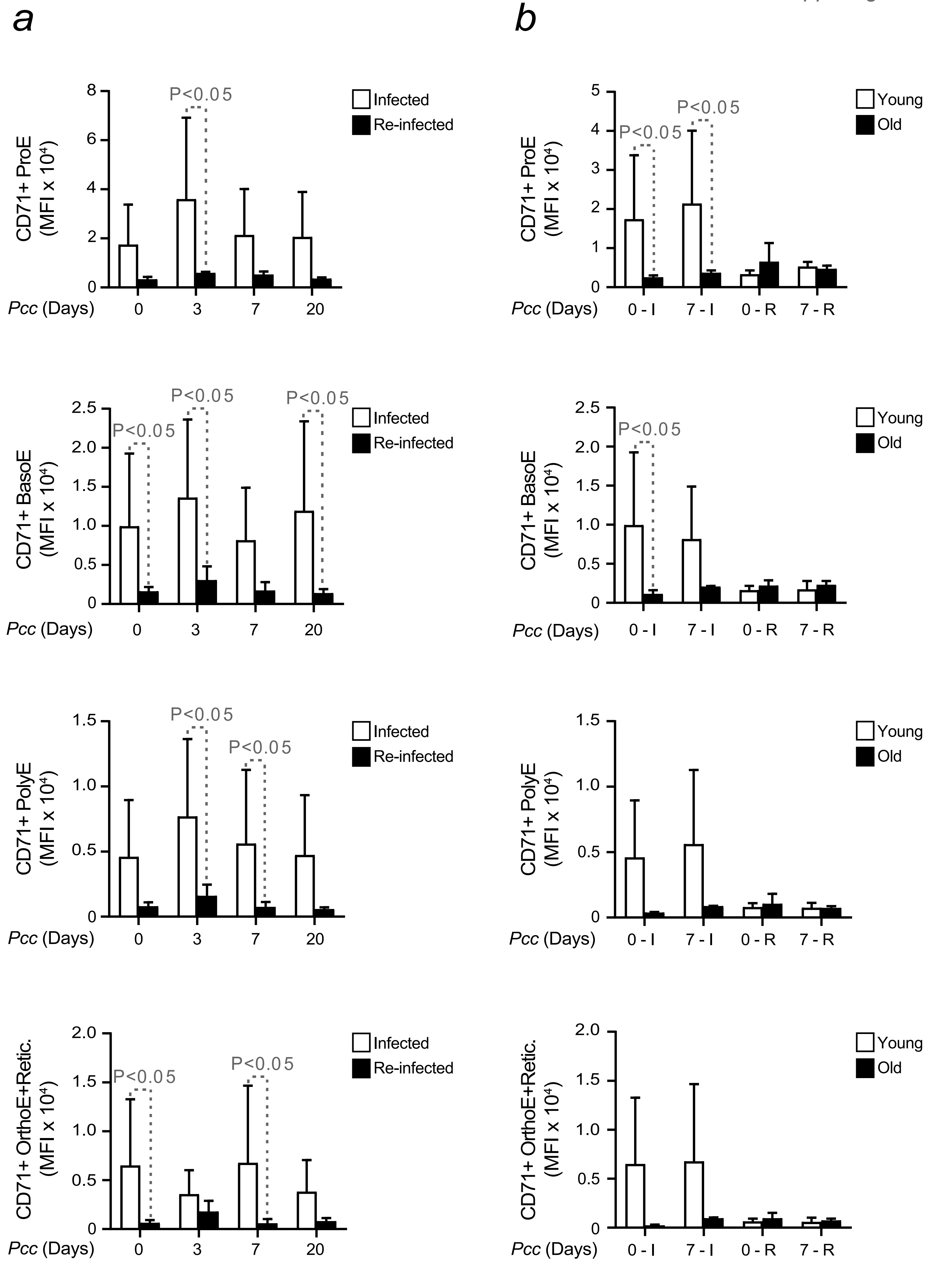
a
b
CD71+ ProE (MFI x 10⁴)
CD71+ BasoE (MFI x 10⁴)
CD71+ PolyE (MFI x 10⁴)
CD71+ OrthoE+Retic. (MFI x 10⁴)
Infected
Re-infected
Young
Old
P<0.05
Pcc (Days)
0
3
7
20
0 - I
7 - I
0 - R
7 - R

a

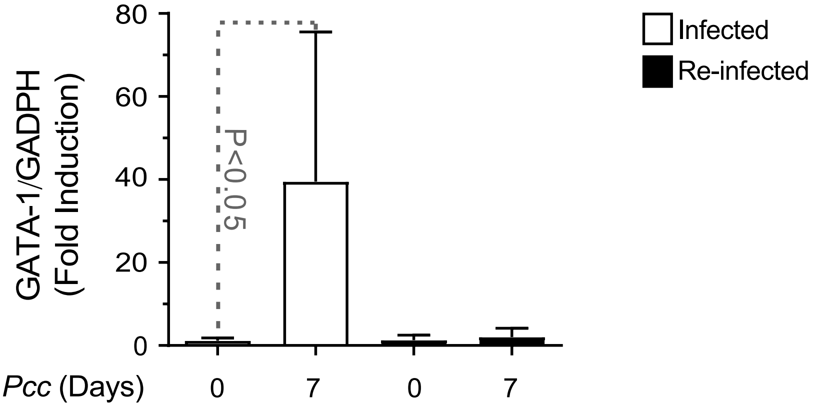
GATA-1/GADPH (Fold Induction)
80
60
40
20
0
P<0.05
Pcc (Days)
0
7
0
7
Infected
Re-infected

b

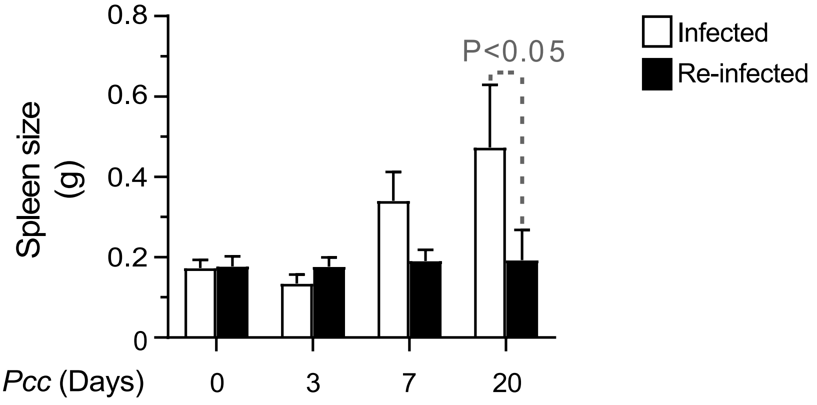
Spleen size (g)
0.8
0.6
0.4
0.2
0
P<0.05
Pcc (Days)
0
3
7
20
Infected
Re-infected

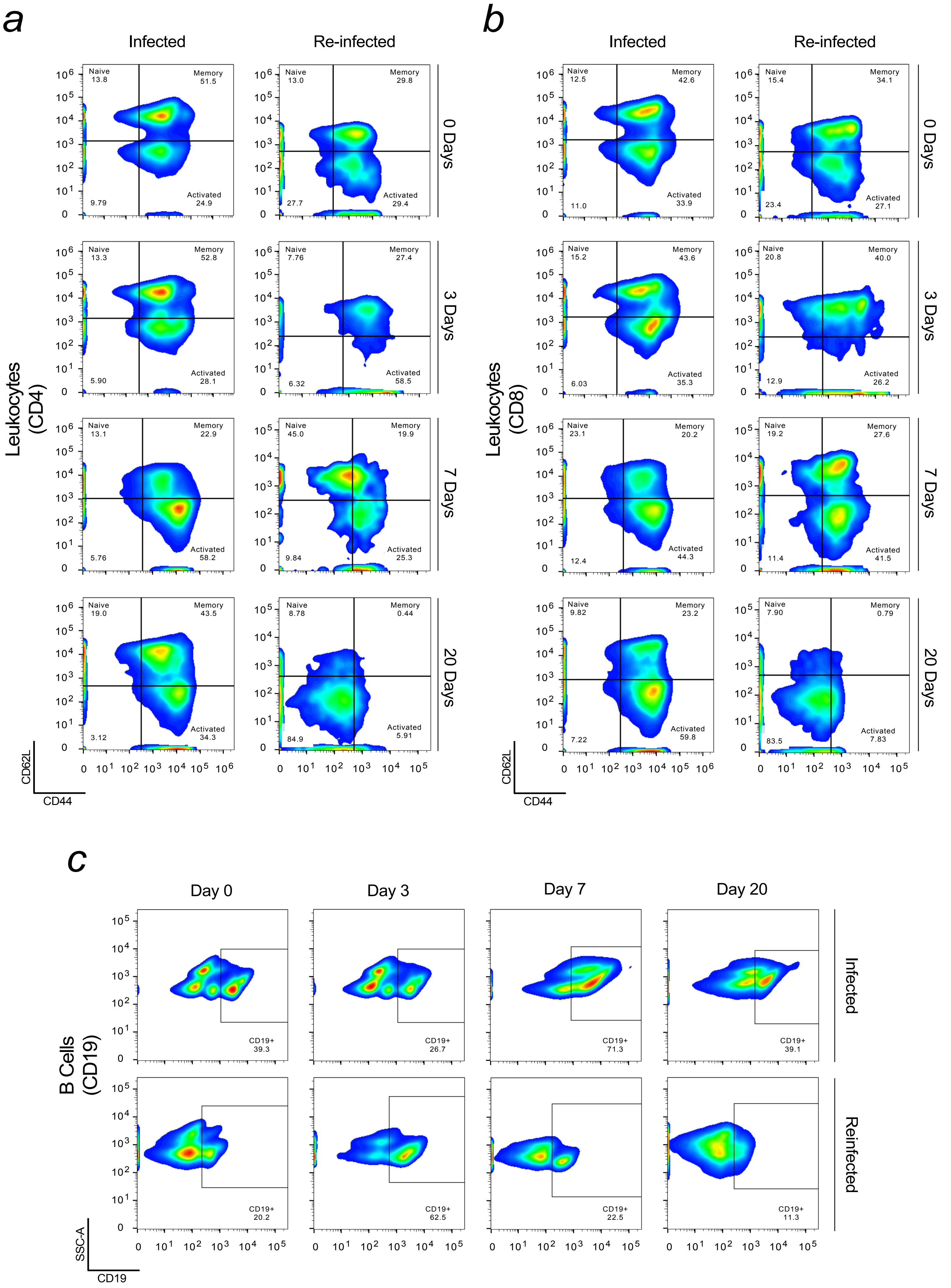
a
Infected
Re-infected
0 Days
3 Days
7 Days
20 Days
Leukocytes (CD4)
Naive 13.8
Memory 51.5
9.79
Activated 24.9
Naive 13.0
Memory 29.8
27.7
Activated 29.4
Naive 13.3
Memory 52.8
5.90
Activated 28.1
Naive 7.76
Memory 27.4
6.32
Activated 58.5
Naive 13.1
Memory 22.9
5.76
Activated 58.2
Naive 45.0
Memory 19.9
9.84
Activated 25.3
Naive 19.0
Memory 43.5
3.12
Activated 34.3
Naive 8.78
Memory 0.44
84.9
Activated 5.91
CD62L
CD44
b
Infected
Re-infected
0 Days
3 Days
7 Days
20 Days
Leukocytes (CD8)
Naive 12.5
Memory 42.6
11.0
Activated 33.9
Naive 15.4
Memory 34.1
23.4
Activated 27.1
Naive 15.2
Memory 43.6
6.03
Activated 35.3
Naive 20.8
Memory 40.0
12.9
Activated 26.2
Naive 23.1
Memory 20.2
12.4
Activated 44.3
Naive 19.2
Memory 27.6
11.4
Activated 41.5
Naive 9.82
Memory 23.2
7.22
Activated 59.8
Naive 7.90
Memory 0.79
83.5
Activated 7.83
CD62L
CD44
c
Day 0
Day 3
Day 7
Day 20
Infected
Reinfected
B Cells (CD19)
CD19+ 39.3
CD19+ 26.7
CD19+ 71.3
CD19+ 39.1
CD19+ 20.2
CD19+ 62.5
CD19+ 22.5
CD19+ 11.3
SSC-A
CD19

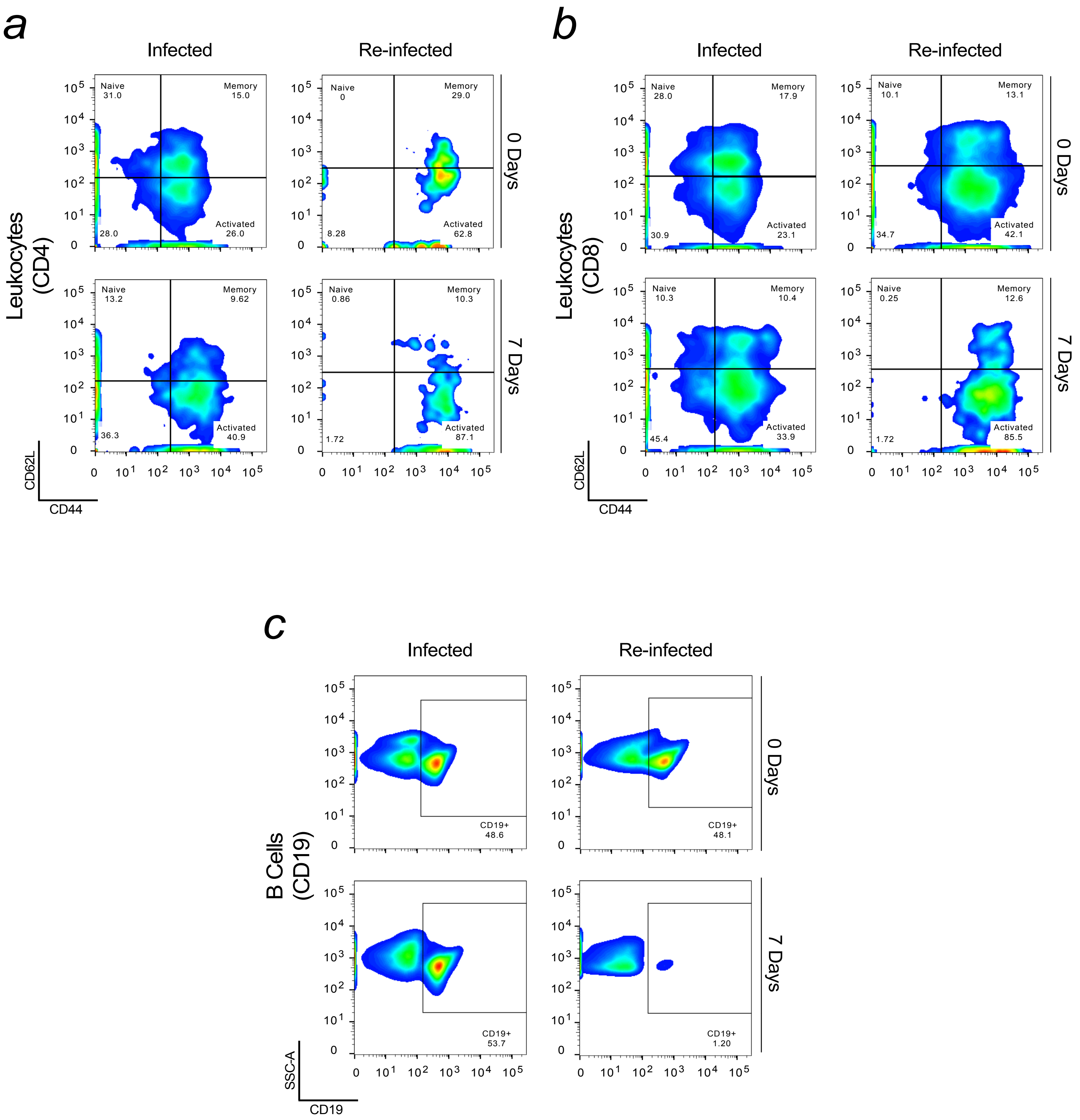
a
Infected
Re-infected
Leukocytes (CD4)
0 Days
7 Days
Naive 31.0
Memory 15.0
28.0
Activated 26.0
Naive 0
Memory 29.0
8.28
Activated 62.8
Naive 13.2
Memory 9.62
36.3
Activated 40.9
Naive 0.86
Memory 10.3
1.72
Activated 87.1
CD62L
CD44
b
Infected
Re-infected
Leukocytes (CD8)
0 Days
7 Days
Naive 28.0
Memory 17.9
30.9
Activated 23.1
Naive 10.1
Memory 13.1
34.7
Activated 42.1
Naive 10.3
Memory 10.4
45.4
Activated 33.9
Naive 0.25
Memory 12.6
1.72
Activated 85.5
CD62L
CD44
c
Infected
Re-infected
B Cells (CD19)
0 Days
7 Days
CD19+ 48.6
CD19+ 48.1
CD19+ 53.7
CD19+ 1.20
SSC-A
CD19

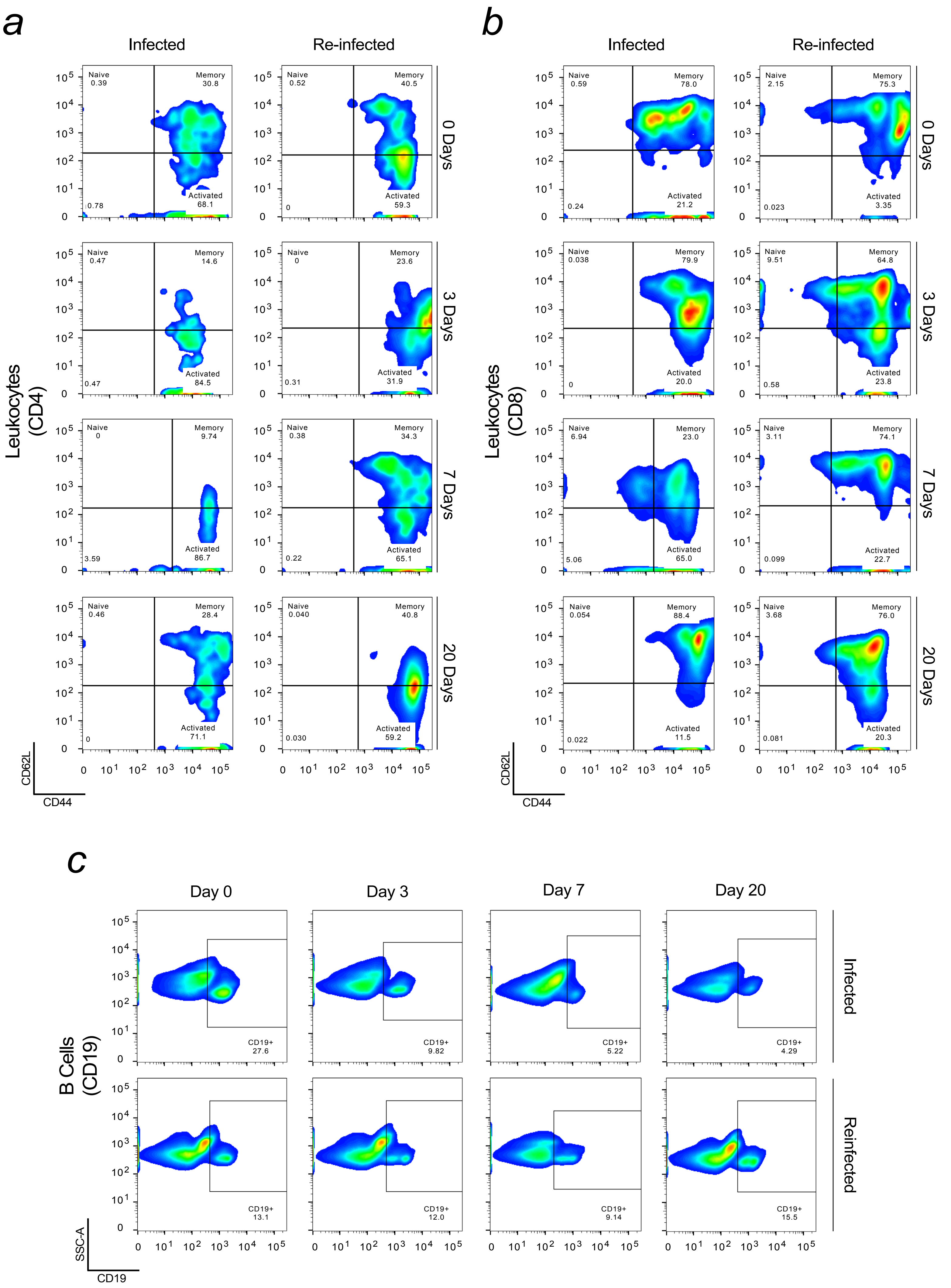
a
Infected
Re-infected
Leukocytes (CD4)
0 Days
3 Days
7 Days
20 Days
Naive 0.39
Memory 30.8
Activated 68.1
0.78
Naive 0.52
Memory 40.5
Activated 59.3
0
Naive 0.47
Memory 14.6
Activated 84.5
0.47
Naive 0
Memory 23.6
Activated 31.9
0.31
Naive 0
Memory 9.74
Activated 86.7
3.59
Naive 0.38
Memory 34.3
Activated 65.1
0.22
Naive 0.46
Memory 28.4
Activated 71.1
0
Naive 0.040
Memory 40.8
Activated 59.2
0.030
CD62L
CD44
b
Infected
Re-infected
Leukocytes (CD8)
0 Days
3 Days
7 Days
20 Days
Naive 0.59
Memory 78.0
Activated 21.2
0.24
Naive 2.15
Memory 75.3
Activated 3.35
0.023
Naive 0.038
Memory 79.9
Activated 20.0
0
Naive 9.51
Memory 64.8
Activated 23.8
0.58
Naive 6.94
Memory 23.0
Activated 65.0
5.06
Naive 3.11
Memory 74.1
Activated 22.7
0.099
Naive 0.054
Memory 88.4
Activated 11.5
0.022
Naive 3.68
Memory 76.0
Activated 20.3
0.081
CD62L
CD44
c
Day 0
Day 3
Day 7
Day 20
B Cells (CD19)
Infected
Reinfected
CD19+ 27.6
CD19+ 9.82
CD19+ 5.22
CD19+ 4.29
CD19+ 13.1
CD19+ 12.0
CD19+ 9.14
CD19+ 15.5
SSC-A
CD19

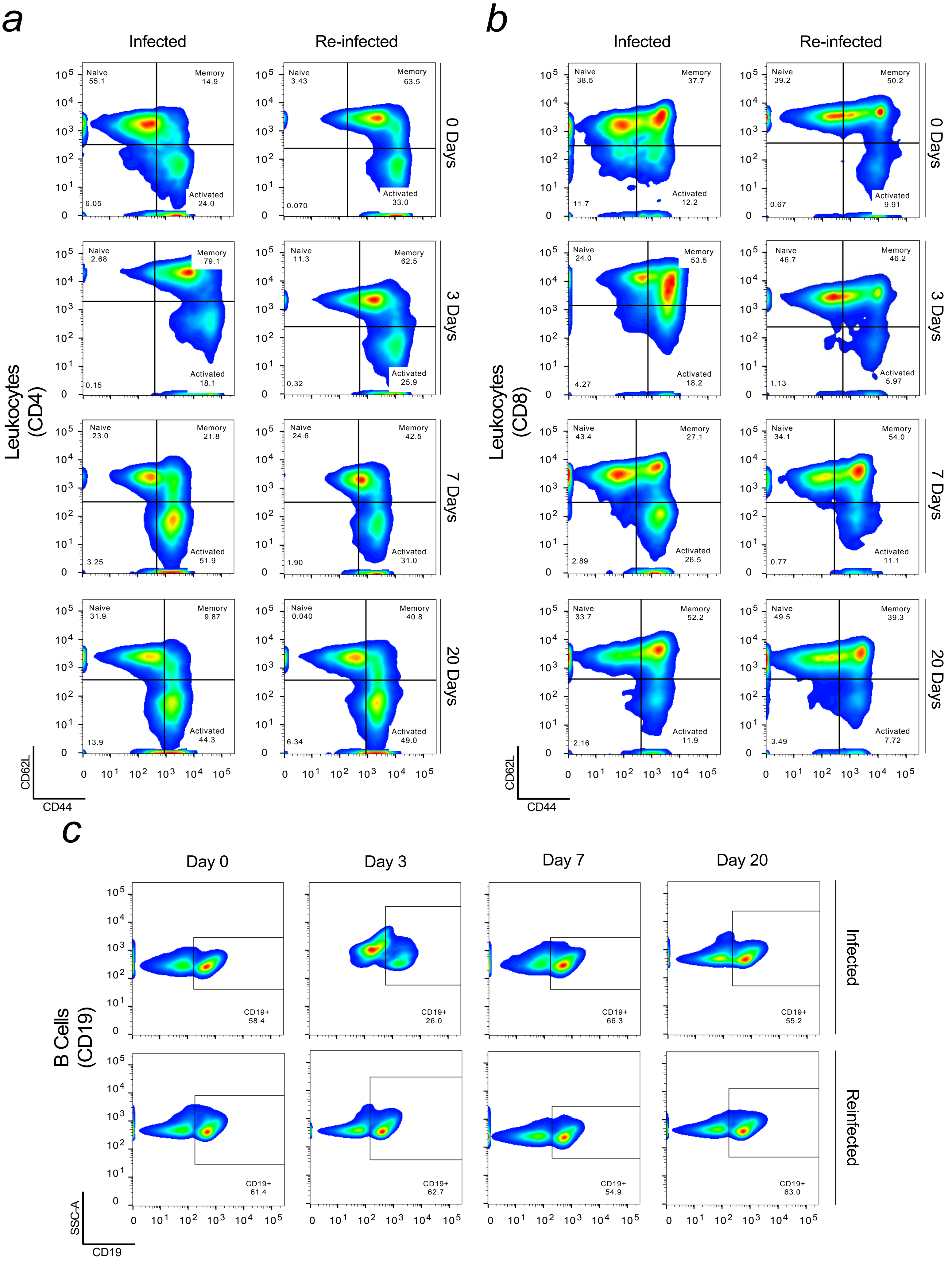
a
Infected
Re-infected
Leukocytes (CD4)
0 Days
3 Days
7 Days
20 Days
Naive 55.1
Memory 14.9
Activated 24.0
6.05
Naive 3.43
Memory 63.5
Activated 33.0
0.070
Naive 2.68
Memory 79.1
Activated 18.1
0.15
Naive 11.3
Memory 62.5
Activated 25.9
0.32
Naive 23.0
Memory 21.8
Activated 51.9
3.25
Naive 24.6
Memory 42.5
Activated 31.0
1.90
Naive 31.9
Memory 9.87
Activated 44.3
13.9
Naive 0.040
Memory 40.8
Activated 49.0
6.34
CD62L
CD44
b
Infected
Re-infected
Leukocytes (CD8)
0 Days
3 Days
7 Days
20 Days
Naive 38.5
Memory 37.7
Activated 12.2
11.7
Naive 39.2
Memory 50.2
Activated 9.91
0.67
Naive 24.0
Memory 53.5
Activated 18.2
4.27
Naive 46.7
Memory 46.2
Activated 5.97
1.13
Naive 43.4
Memory 27.1
Activated 26.5
2.89
Naive 34.1
Memory 54.0
Activated 11.1
0.77
Naive 33.7
Memory 52.2
Activated 11.9
2.16
Naive 49.5
Memory 39.3
Activated 7.72
3.49
CD62L
CD44
c
Day 0
Day 3
Day 7
Day 20
B Cells (CD19)
Infected
Reinfected
CD19+ 58.4
CD19+ 26.0
CD19+ 66.3
CD19+ 55.2
CD19+ 61.4
CD19+ 62.7
CD19+ 54.9
CD19+ 63.0
SSC-A
CD19

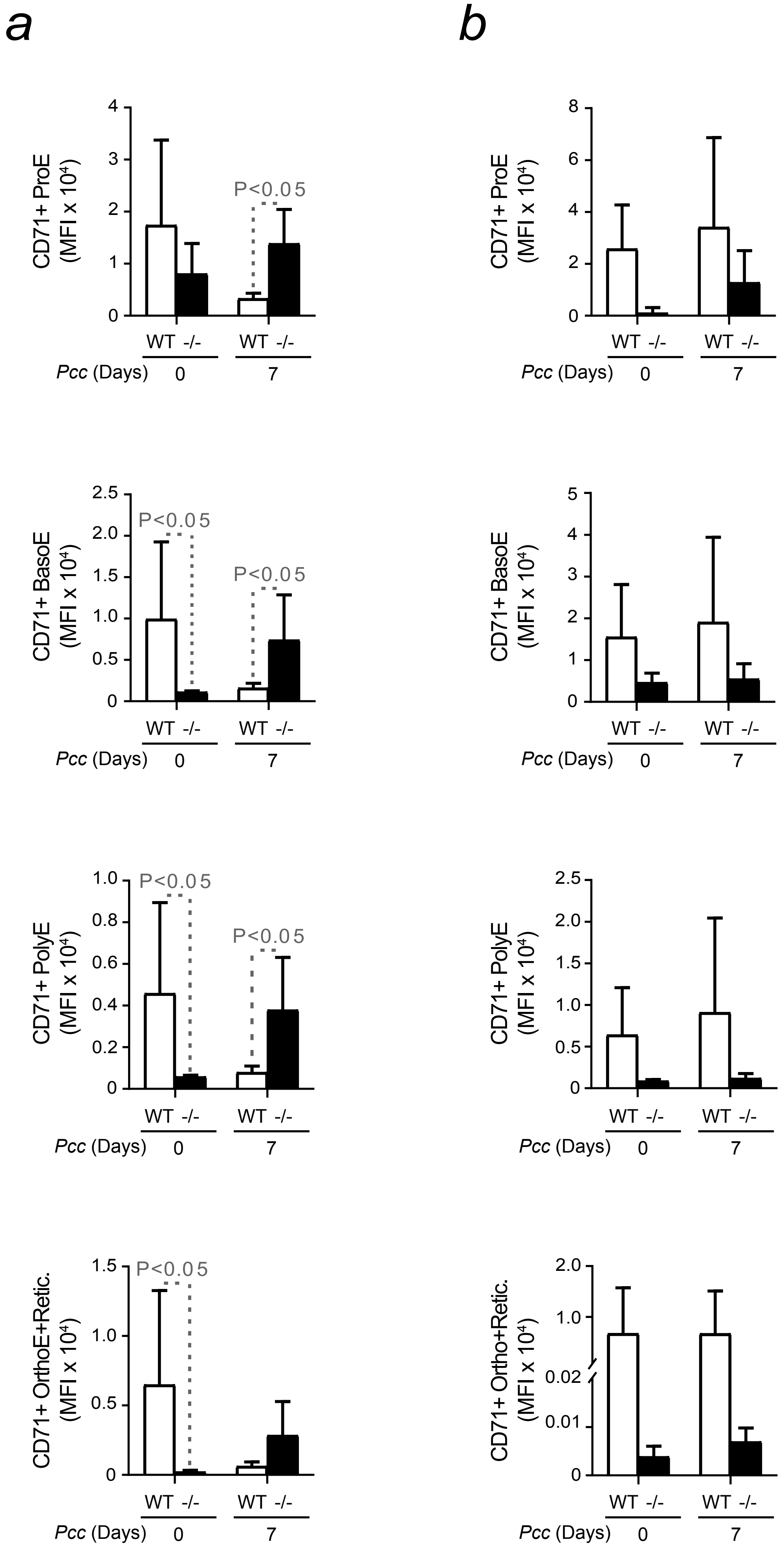
a
b
CD71+ ProE (MFI x 10⁴)
CD71+ BasoE (MFI x 10⁴)
CD71+ PolyE (MFI x 10⁴)
CD71+ OrthoE+Retic. (MFI x 10⁴)
CD71+ Ortho+Retic. (MFI x 10⁴)
P<0.05
WT -/-
WT -/-
Pcc (Days) 0 7

Supplementary Figure legends

Suppl. Fig. S1 – CD71 assessment in erythroid populations of *Pcc* Infected and Reinfected young vs. aged mice. Flow cytometry was used to study the abundance of CD71 in erythroid cells in bone marrow of *Pcc* infected and reinfected mice at days 3, 7, and 20. The non-manipulated group was compared to recovered animals (R, day 60 post-first infection). (a) Histogram representations of the number of CD71+ cells in erythroid proerythroblast (ProE), basophilic (BasoE), polychromatic (PolyE), and orthochromatic and reticulocytes (OrthoE+Retic), collected from the bone marrow of young (8-12 weeks) mice. (b) Histogram representations of the number of CD71+ cells in erythroid proerythroblast (ProE), basophilic (BasoE), polychromatic (PolyE), and orthochromatic and reticulocytes (OrthoE +Retic), collected from the bone marrow of young (8-12 weeks) and old (52-60 weeks) infected and reinfected mice, at indicated time points. Data were presented as mean values ± standard deviations (n=8-13). Statistically significant differences were calculated by one-way ANOVA and indicated accordingly.

Suppl. Fig. S2 – CD71 assessment in erythroid populations of *Pcc* Infected and Reinfected young vs. aged mice. Flow cytometry was used to study the median fluorescen intensity (MFI) of CD71 in erythroid cells in bone marrow of *Pcc* infected and reinfected mice at days 3, 7, and 20. The non-manipulated group was compared to recovered animals (R, day 60 post-infection). (a) Histogram representations of the number of CD71+ cells in erythroid proerythroblast (ProE), basophilic (BasoE), polychromatic (PolyE), and orthochromatic and reticulocytes (OrthoE +Retic), collected from the bone marrow of young (8-12 weeks) mice. (b) Histogram representations show the MFI of CD71+ cells in erythroid proerythroblast (ProE), basophilic (BasoE), polychromatic (PolyE), and orthochromatic and reticulocytes (OrthoE+Retic), collected from the spleen of young (8-12 weeks) and old (52-60 weeks) infected and reinfected mice, at indicated time points. Data were presented as mean values ± standard deviations (n=8-13). Statistically significant differences were calculated by one-way ANOVA and indicated accordingly.

Suppl. Fig. S3 – Assessment of *GATA-1* expression in the spleen and organ size in infected and reinfected mice. (a) *GATA-1* mRNA expression, quantified by qRT-PCR, in spleen samples of infected and reinfected mice, at days 0, 3, 7 and 20 post-*Pcc* inoculation. (b). Spleen size, measured in grams (g), upon harvesting from infected and reinfected mice, at indicated time points. Data were presented as mean values ± standard deviations (n=8-13). Statistically significant differences were calculated by one-way ANOVA and indicated accordingly.

Suppl. Fig. S4 – T and B immune cell activation in the blood of infected and reinfected mice. Gating strategy of freshly isolated blood immune cells, at days 0, 3, 7 and 20 post-*Pcc* inoculation. (a) CD4+; (b) CD8+ T cells. The activation of T cell subtypes was determined through the expression of CD44 and CD62L, allowing to identify naïve (CD44–CD62L+), effector or activated (CD44+CD62L–), and memory cells (CD44+CD62L+). (c) Circulating B cells (CD19+). Data are expressed as mean values ± standard deviations (n=7-12). Statistically significant differences were calculated by one-way ANOVA and indicated accordingly.

Suppl. Fig. S5 – T and B immune cell activation in the blood of infected and reinfected young vs. old mice. Gating strategy of freshly isolated blood immune cells, at days 0 and 7 post-*Pcc* inoculation, from young (8-12 weeks old) and aged (52-60 weeks old) mice: (a) CD4+; (b) CD8+ T cells. The activation of T cell subtypes was determined through the expression of CD44 and CD62L, allowing to identify naïve (CD44–CD62L+), effector or activated (CD44 +CD62L–), and memory cells (CD44+CD62L+). (c) Circulating B cells (CD19+). Data are expressed as mean values ± standard deviations (n=7-10). Statistically significant differences were calculated by one-way ANOVA and indicated accordingly.

Suppl. Fig. S6 – T and B immune cell activation in the bone marrow of infected and reinfected mice. Gating strategy of freshly isolated bone marrow immune cells, at days 0, 3, 7 and 20 post-*Pcc* inoculation. (a) CD4+; (b) CD8+ T cells. The activation of T cell subtypes was determined through the expression of CD44 and CD62L, allowing to identify naïve (CD44–CD62L+), effector or activated (CD44+CD62L–), and memory cells (CD44+CD62L+). (c) Circulating B cells (CD19+). Data are expressed as mean values ± standard deviations (n=7-12). Statistically significant differences were calculated by one-way ANOVA and indicated accordingly.

Suppl. Fig. S7 – T and B immune cell activation in the spleen of infected and reinfected mice. Gating strategy of freshly isolated spleen immune cells, at days 0, 3, 7 and 20 post-*Pcc* inoculation. (a) CD4+; (b) CD8+ T cells. The

activation of T cell subtypes was determined through the expression of CD44 and CD62L, allowing to identify naïve (CD44–CD62L+), effector or activated (CD44+CD62L–), and memory cells (CD44+CD62L+). (c) Circulating B cells (CD19+). Data are expressed as mean values ± standard deviations (n=7-12). Statistically significant differences were calculated by one-way ANOVA and indicated accordingly.

Suppl. Fig. S8.– CD71 assessment in erythroid populations of *Pcc* Infected wild-type and Rag2-deficient mice. Flow cytometry was used to study the median fluorescen intensity (MFI) of CD71 in erythroid cells in bone marrow of *Pcc* infected wild-type and Rag2-knockout mice at day 7. (a) Histogram representations of the number of CD71+ cells in erythroid proerythroblast (ProE), basophilic (BasoE), polychromatic (PolyE), and orthochromatic and reticulocytes (OrthoE+Retic), collected from the bone marrow. (b) Histogram representations show the MFI of CD71+ cells in erythroid proerythroblast (ProE), basophilic (BasoE), polychromatic (PolyE), and orthochromatic and reticulocytes (OrthoE+Retic), collected from the spleen. Data were presented as mean values ± standard deviations (n=8-13). Statistically significant differences were calculated by one-way ANOVA and indicated accordingly.
